# Non-invasive systemic viral delivery of human alpha-synuclein mimics selective and progressive neuropathology of Parkinson’s disease in rodent brains

**DOI:** 10.1186/s13024-023-00683-8

**Published:** 2023-11-27

**Authors:** Morgan Bérard, Laura Martínez-Drudis, Razan Sheta, Omar M. A. El-Agnaf, Abid Oueslati

**Affiliations:** 1 CHU de Québec Research Center, Axe Neurosciences, Quebec City, Canada; 2 Department of Molecular Medicine, Faculty of Medicine, Université Laval, Quebec City, Canada; 3 Neurological Disorders Research Center, Qatar Biomedical Research Institute, Hamad Bin Khalifa University, Qatar Foundation, Doha, 34110 Qatar

**Keywords:** Alpha-synuclein, Adeno-associated virus, AAV-PHP.eB, Neurodegeneration, Parkinson’s Disease, Animal model, Systemic delivery, PD-like symptoms, Genetic model of PD, Protein aggregation.

## Abstract

**Background:**

Alpha-synuclein (α-syn) aggregation into proteinaceous intraneuronal inclusions, called Lewy bodies (LBs), is the neuropathological hallmark of Parkinson’s disease (PD) and related synucleinopathies. However, the exact role of α-syn inclusions in PD pathogenesis remains elusive. This lack of knowledge is mainly due to the absence of optimal α-syn-based animal models that recapitulate the different stages of neurodegeneration.

**Methods:**

Here we describe a novel approach for a systemic delivery of viral particles carrying human α-syn allowing for a large-scale overexpression of this protein in the mouse brain. This approach is based on the use of a new generation of adeno-associated virus (AAV), AAV-PHP.eB, with an increased capacity to cross the blood-brain barrier, thus offering a viable tool for a non-invasive and large-scale gene delivery in the central nervous system.

**Results:**

Using this model, we report that widespread overexpression of human α-syn induced selective degeneration of dopaminergic (DA) neurons, an exacerbated neuroinflammatory response in the substantia nigra and a progressive manifestation of PD-like motor impairments. Interestingly, biochemical analysis revealed the presence of insoluble α-syn oligomers in the midbrain. Together, our data demonstrate that a single non-invasive systemic delivery of viral particles overexpressing α-syn prompted selective and progressive neuropathology resembling the early stages of PD.

**Conclusions:**

Our new in vivo model represents a valuable tool to study the role of α-syn in PD pathogenesis and in the selective vulnerability of nigral DA neurons; and offers the opportunity to test new strategies targeting α-syn toxicity for the development of disease-modifying therapies for PD and related disorders.

**Supplementary Information:**

The online version contains supplementary material available at 10.1186/s13024-023-00683-8.

## Background

Parkinson’s disease (PD) is a neurodegenerative disorder characterized by a massive and progressive neuronal loss, essentially the dopaminergic neurons of the midbrain, and the presence of intraneuronal proteinaceous inclusions, called Lewy bodies (LBs) in the surviving neurons [1, 2]. A tilting point in the field of PD was the discovery that LBs are mainly constituted of the aggregated form of a presynaptic protein, alpha-synuclein (α-syn) [3–5]. Subsequent genetic studies reported a link between mutations or multiplications of *SNCA*, the gene coding for α-syn, and the manifestation of familial forms of PD [6–12]. Together, these findings pointed to α-syn as a key player in PD pathogenesis. However, the exact role of this protein in neurodegeneration is not yet fully elucidated [13]. To address this question, growing efforts have been made to create experimental models of PD based on α-syn overexpression to mimic α-syn abnormal accumulation in rodent brains. The first approach was the use of transgenic (Tg) mice overexpressing α-syn under different promoters (human platelet-derived factor, Thy1, BAC) [14, 15]. Unfortunately, only few Tg models exhibited dopaminergic neuronal loss, and at very late stages of the disease manifestation (8–9 months) [14], while other did not report neuronal loss, probably due to some developmental compensatory mechanisms [15]. An alternative approach consisted in using Adeno-associated viral (AAV) delivery system to directly overexpress α-syn in the midbrain of adult rodents [16, 17]. Although this approach allowed for the induction of a significant dopaminergic neuronal loss in the midbrain [18–20], it presented with several limitations, notably the temporal and spatial restriction of α-syn overexpression and the need for high levels of technical expertise and the use of specific stereotaxic equipment [21].

More recently, a new generation of AAV particles, AAV-PHP.eB, has been created, with an increased capacity to cross the blood-brain barrier (BBB), offering a viable tool for a large-scale gene delivery in the central nervous system (CNS) when administered systemically [22]. In addition to being less invasive than the stereotaxic intracerebral injection, this technology led to stable transgene expression and the co-transduction of different brain regions [22, 23], thus providing a potential useful tool for a rapid and large-scale overexpression of α-syn in the adult brain and the creation of an in vivo model of PD.

Taking advantage of this new viral strain, we overexpressed human α-syn (hα-syn) in the mouse brain and assessed the impact of such overexpression on the neuronal loss in different brain regions and the manifestation of PD-like symptoms. First, we confirmed the expression of α-syn in different brain regions and accumulation in the midbrain. Then, we observed that systemic hα-syn overexpression induced a selective dopaminergic neuronal loss in the midbrain, the induction of PD-like symptoms, the accumulation of pathological α-syn, and a neuroinflammatory reaction in the midbrain. Collectively, our findings report on the description of a new α-syn-based rodent model of PD, offering a valuable tool to decorticate the cellular and molecular mechanisms of PD pathogenesis and to develop efficient disease-modifying treatments for synucleinopathies.

## Methods

### **Plasmid construction and production of recombinant adeno-associated 2/ PHP.eB viral vectors**

pAAV-CAG-mTurquoise2 plasmid was kindly provided by Dr. Viviana Gradinaru (Addgene plasmid # 99,122). pcDNA-human α-syn plasmid was kindly provided by Dr. Hilal Lashuel (EPFL, Switzerland). To generate pAAV-CAG-human α-syn plasmid (mTurquoise2 was replaced by human α-syn). For the production of adeno-associated viral (AAV) vectors, the cDNA encoding human α-syn was sub cloned in pAAV-CAG-mTurquoise2 using standard cloning procedures and verified by sequencing. The production of the recombinant pseudotyped AAV2/PHP.eB vectors (serotype 2 genome/PHP.eB capsid) and relative infectivity titers were performed by the Canadian Neurophotonics Platform (CERVO, Quebec City). The final viral titers were: 1.0 × 10^13^ GC/ml for AAV-CAG-mTurquoise2 and 3.0 × 10^12^ GC/ml for AAV-CAG-human α-syn.

### Intra veinous injections of AAV viral particles

Three-month old C57/BL6N male mice were obtained from Charles River Laboratories and habituated during a 7-day period before any handling. Mice were housed on 12 h light/dark cycles with *ad libitum* access to food and water. All animal experiments were approved by the Animal Welfare Committee of Université Laval in accordance with the Canadian Council on Animal Care policy. Mice were anesthetized with 2% isoflurane-O^2^ and received retro orbital injection of 50 µL PBS (pH 7.4) only (control group), or the equivalent volume of PBS containing 10^11^ GC of either AAV-CAG-mTurquoise2 (mTurq group) or AAV-CAG-human α-syn (hα-syn group). For each group, mice were sacrificed 15 and 90 days post-AAV injection.

### Behavioral tests

All behavioral tests were completed during the light phase of the light-dark cycle between 8 am and 4 pm. Mice were habituated to the experimenter and the testing room for several days prior to the start of testing. All group assignments were randomized, and the experimenter was blinded to the received viral vector treatment during the testing.

#### The cylinder test

The cylinder test was performed to evaluate the motor impairment induced after a dopaminergic neuronal loss, by quantifying the deficits in using the contralateral forelimb (akinesia) [24–26]. Briefly, mice were placed in a transparent Plexiglas cylinder (15 cm diameter, 12 cm high) surrounded by a mirror to monitor the mouse from all angles and videotaped using a camera (Microsoft LifeCam Cinema) (H5D-00018; Microsoft, Redmond, WA, U.S). A total number of 30 forepaw contacts made on the cylinder wall by the ipsilateral or the contralateral (impaired) forelimbs were scored, and the results were expressed as the ratio of contralateral contacts relative to the total contacts made by both forelimbs.

#### The grip force test

Grip Strength (CHATILLON® DFE Series) (Ametek Inc. Berwyn, PA, USA) was used to measure the muscle strength of forelimbs as previously described [27]. During testing, the mouse is placed horizontally on the grid to allow gripping of the grid with the forepaws while being supported by the tail. Once the grid is gripped, the mouse is pulled back until the grip on the grid is released and the value of the grip strength on the apparatus is recorded. Mice underwent three trials per testing session and analysis was performed on the average of the three trials.

#### The rotarod test

The rotarod test was used to measure motor coordination, endurance, and balance [28]. All mice were pre-trained on the rotarod (LE 8200; Panlab Harvard apparatus, Holliston, MA, USA) at baseline to reach a stable performance. At testing, mice were placed on the rotarod for three consecutive 3-min trials, at the fixed speed of 12 RPM with 1 min of rest between each trial. The latency to fall was recorded for each trial, and the mean value from each speed and was used for the analysis.

#### The gait test

To test for gait abnormalities, footprint gait analysis was performed as previously described with some modifications [29]. Briefly, the hind- and forefeet of the mice were painted with blue (right paws) and orange (left paws) non-toxic paint (Liquid tempera) (SCHOLA, Marieville, QC, Canada) immediately prior to placement in a 45 cm long and 15 cm wide runway coated in newsprint paper. Stride length was measured as the distance of forward movement between paw prints. The mean value of each set of three values measuring stride length was used in the analysis.

#### Y-Maze test

Spatial working memory was evaluated by spontaneous alternation task using a symmetrical Y-maze made of black Plexiglas. The three equal-sized arms measured 30 cm long, 15 cm high and 8 cm wide. The Y-maze was located on a white wood platform in a dimly illuminated room. We followed the procedure previously described [30, 31]. Mice were placed in the central area of the maze, a neutral zone, and were allowed to freely explore during 5 min. After each animal session, the maze and the platform were cleaned with alcohol. We recorded the sequence and the total number of arm entries. Arm entrance was only counted when the animal completely introduced its whole body into a specific arm. Collected data were analyzed in terms of percentage of correct alternations. This percentage was calculated as the number of overlapping triads containing entries into all three arms divided by the maximum possible alternations (the total number of arm entries minus 2) x 100. As the re-entries into the same arm were not considered for the analysis, the probability to choose correctly between the arm visited on the step before (non-alternation) and the one visited less recently (alternation) was 50%. The analysis performed was based on the percentage of spontaneous alternation.

#### Morris water maze (MWM)

Spatial learning and memory were measured by the MWM test that consisted of a circular pool (78 cm diameter, walls 40 cm high) with water made opaque with white non-toxic water-based tempera paint in which mice were trained to escape from the water by swimming to an escape platform as previously described [32]. Water temperature was maintained at 25 ± 0.5 °C.

The pool was divided into four quadrants (cardinal points: NE, NW, SW and SE) using the video tracking software ANY-maze (Version 4.8; Stoelting Co., Wood Dale, Il). The platform was placed in the center of one quadrant of the pool, 15 cm from the pool’s edge and submerged 1 cm beneath the water surface.

The spatial acquisition phase consisted of 12 training trials: 4 training trials per day and 3 training days with an inter-trial interval of 1 h. Mice were released randomly with their heads facing the pool wall from the four cardinal points and allowed to swim and search for the platform for 60 s. If mice did not locate the platform after 60 s, animals were manually placed on the platform and allowed to remain on it for 15 s. The platform and the visual cues remained in the same position throughout the training trials. The latency and distance to reach the hidden platform were recorded. For the analysis, the mean per training day was obtained for each parameter.

One day after the training phase, subjects received a probe trial, in which the platform was removed while the visual clues around the pool remained. Mice were released from the cardinal point opposite to the target quadrant (quadrant in which the hidden platform was located during the training trials) and were allowed to swim freely for 60 s. The time and distance traveled in each quadrant was recorded for the analysis.

### Tissue processing and immunohistological analysis

For tissue processing, animals were sacrificed with ketamine/xylazine overdose (90 mg/ml/10 mg/ml, injected 0.1 ml/30 g mice). Brains were collected after transcardial perfusion with PBS containing 1% Phosphatase/Proteinase inhibitors. The hemispheres were separated, one was rapidly frozen on dry ice and stored at -80 °C and the other was post-fixed for 36 h in 4% PFA, then incubated in 20% sucrose-PBS 2 times for 24 h. After post-fixation, brains were cut in coronal Sect. (30 μm thick) with a microtome (SM2000R; Leica, Wetzlar, Germany), and slices were stored at -20 °C in cryoprotection medium.

For immunohistochemistry, slices were washed 3 times with PBS (10 min) and then incubated for 1 h at room temperature (RT) in blocking buffer (3% bovine serum albumin (BSA), 0.1% Triton X100- PBS) and then incubated overnight at 4 °C with primary antibodies prepared in blocking buffer (see Table 1). For enzymatic revelation, slices were incubated with biotinylated secondary antibody (see Table 1). Slices were washed 3 times with PBS (10 min). The VECTASTAIN Elite ABC Kit (PK6100; Vectastain, Burlingame, CA) was then used for enzymatic staining. Slices were incubated in 3,3’-diaminobenzidine tetrahydrochloride (DAB) (D5905; Sigma-Aldrich, St. Louis, MO, USA), and subsequently counterstained with Cresyl Violet acetate stain (Nissl) (C5042; Sigma-Aldrich, St. Louis, MO, USA). For microscopy imaging, slices were mounted on Superfrost Plus microscope slides (12-550-15; Fisherbrand, Waltham, MA, USA), dried and finally mounted in DTX Mounting Medium (13,512; EMS, Hatfield, PA, USA).

**Table 1:** List of the antibodies used in this study

Antigen/species	Antibody name /catalog number	Epitope	Concentration immunoblotting	Concentration IHC or IF	Source
Primary antibodies					
α-syn/ mouse	Syn1 / 610,787	15–123	1:1000	1:1000	BD Laboratory (Billerica, MA, USA)
Human α-syn/ mouse	LB509 / 180,215	115–122		1:500	ThermoFisher Scientific (Waltham, MA, USA)
α-syn/ rabbit	Syn FL-140 / sc-10,717	65–91	1:1000		Santa Cruz Biotech (Dallas, TX, USA)
α-syn/ rabbit	Anti-Alpha-synuclein antibody [MJFR1]/ (ab138501)	118–123	1:1000		Abcam (Cambridge, UK)
pS129 α-Syn/ mouse	WAKO / pSyn #64	Phospho-Ser129	1:1000	1:2000	WAKO (Richmond, VA, USA)
pS129 α-Syn/rabbit	Anti-Alpha-synuclein (phospho S129) antibody [EP1536Y]/ Ab51253	Phospho-Ser129	1:1000		Abcam (Cambridge, UK)
pS129 α-Syn/mouse	pS129 (Ghanem et al., 2022)	Phospho-Ser129	100 ng/ml		Ghanem et al., 2022
Beta-actin/ mouse	β-actin clone BA3R / G043	Beta-actin N-terminal peptide-KLH conjugates.	1:10000		Abm (Vancouver, BC, Canada)
Drebrin/mouse	Drebrin clone MX823/612,128	C-terminal 632–649 coupled to KLH	1:1000		Progen (Heidelberg, Germany)
GAPDH/ mouse	GAPDH loading control/G041	–	1:2500		Abm (Vancouver, BC, Canada)
GFP/ rabbit	GFP/A-6455	Full length protein	1:5000		ThermoFisher Scientific (Waltham, MA, USA)
Non-phosphorylated α-syn/ mouse	4B1	125–133	100 ng/ml		Ghanem et al., 2022
Full-length α-syn aggregates /mouse	Syn-O2		100 ng/ml		Ghanem et al., 2022
Full-length α-syn aggregates /mouse	Syn-O1		100 ng/ml		Ghanem et al., 2022
Full-length α-syn aggregates /mouse	Syn-O3		100 ng/ml		Ghanem et al., 2022
Full-length α-syn aggregates /mouse	Syn-F1		100 ng/ml		Ghanem et al., 2022
Full-length α-syn aggregates /mouse	Syn-F2		100 ng/ml		Ghanem et al., 2022
GFAP/ rabbit	anti-Glial Fibrillary Acidic Protein / Z0334	Full length protein		1:800	Dako (Santa Clara, CA, USA)
GFAP/ mouse	Anti-Glial Fibrillary Acidic Protein clone GA5/ MAB360	Full length protein		1:800	Millipore (Temecula, CA, USA)
Iba1/ rabbit	Anti-Ionized calcium binding adaptor molecule 1/ 019-19741	Synthetic peptide (Iba1 C-terminal sequence)		1:750	WAKO (Richmond, VA, USA)
mCherry/ rabbit	Anti-mCherry antibody/ AB167453	mCherry	1:1000	1:1000	Abcam (Cambridge, UK)
NeuN/ mouse	Anti-NeuN Antibody, clone A60 / MAB377			1:1000	Millipore (Temecula, CA, USA)
PSD95/mouse	PSD95 clone K28/43/75–028	77–299 (PDZ domains 1 and 2)	1:1000 UC		Davis/NIH NeuroMab Facility (Davis, CA, USA)
TH/ mouse	Anti-Tyrosine Hydroxylase clone LNC1 /MAB318	Recognizes an epitope on the outside of the regulatory N-terminus		1:1000	Millipore (Temecula, CA, USA)
Synpato-physin/ rabbit	Synaptophysin/PA1–1043	253–272 1:50000–	1:25000		Invitrogen (Waltham, MA, USA)
Secondary antibodies					
IRDye 680RD Goat anti-Rabbit IgG Secondary Antibody	680RD-conjugated goat anti-rabbit/ 926–68,071		1:20000		LI-COR Biosciences (Lincoln, NE, USA)
IRDye 800CW Goat anti-Rabbit IgG Secondary Antibody	800CW-conjugated goat anti-rabbit/ 926–32,211		1:20000		LI-COR Biosciences (Lincoln, NE, USA)
IRDye 680RD Goat anti-Mouse IgG Secondary Antibody	680RD-conjugated goat anti-mouse/ 926–68,070		1:20000		LI-COR Biosciences (Lincoln, NE, USA)
IRDye 800CW Goat anti-Mouse IgG Secondary Antibody	800CW-conjugated goat anti-mouse/ 926–32,210		1:20000		LI-COR Biosciences (Lincoln, NE, USA)
Goat Anti-Mouse IgG Antibody (H + L), Biotinylated	Biotinylated Goat Anti-mouse/ BA-9200			1:500	Vector Laboratories (Burlingame, CA, USA)
Alexa Fluor 488 goat anti-rabbit (H + L)	Alexa Fluor 488 goat anti-rabbit/ A-11,008			1:1000	Invitrogen (Waltham, MA, USA)
Alexa Fluor 633 goat anti-rabbit (H + L)	Alexa Fluor 633 goat anti-rabbit/ A21071			1:1000	Invitrogen (Waltham, MA, USA)
Alexa Fluor 488 goat anti-mouse (H + L)	Alexa Fluor 488 goat anti-mouse/ A-11,029			1:1000	Invitrogen (Waltham, MA, USA)
Alexa Fluor 633 goat anti-mouse (H + L)	Alexa Fluor 633 goat anti-mouse/ A-21,052			1:1000	Invitrogen (Waltham, MA, USA)
Cy™3 AffiniPure Goat Anti-Rabbit IgG (H + L)	111-165-003			1:1000	Jackson ImmunoResearch Laboratories, Inc 872 West Baltimore Pike West Grove, Pennsylvania 19,390 USA

For immunofluorescence, slices were washed 3 times with PBS (10 min) and then incubated for 1 h at RT in blocking buffer (3% BSA, 0.1% Triton X100- PBS) and then incubated overnight at 4 °C with primary antibodies prepared in blocking buffer (see Table 1). The slices were then washed 3 times with PBS (10 min) and incubated for 2 h at RT with the appropriate secondary antibodies in 0.1% Triton X100-PBS conjugated to Alexa Fluor-488 or Alexa Fluor-633 (see Table 1). After secondary antibody incubation, slices were washed 3 times with 0.1% Triton X100- PBS (10 min), then incubated in DAPI (1:5000 for 7 min) and finally washed 2 times with PBS (10 min). For microscopy imaging, slices were mounted on Superfrost Plus microscope slides in Fluoromount-G T (17984-25; EMS, Hatfield, PA, USA) mounting media and were left to dry in the dark for 2 days.

### Unbiased stereological estimation of neurons in ROIs

Neuron number was estimated using unbiased stereology according to the optical fractionator principle described by West et al., [33]. Briefly, we performed the immunofluorescence protocol as described above with NeuN primary antibody (MAB377) and Cy3 corresponding secondary antibody (AB_2338000). The number of NeuN^+^ cells were determined by evaluating every fourth coronal Sect. (1/4) covering the regions of interest (ROIs): striatum, motor cortex, hippocampus, and substantia nigra. The ROIs were delineated at low magnification (4X) and then the NeuN^+^ cells were counted at magnification (20X). NeuN^+^ cells were counted in a blinded fashion and the results were expressed as the mean ± standard error of the total number of NeuN^+^ cells in each structure. Analysis was performed using the Stereologer software (SRC Biosciences, FL, USA) on epifluorescence microscope equipped with Axiocam monochrome camera as described [29]. The parameters used for the stereological analysis were as follows: grid size, 450 × 450 μm; counting frame, 180 × 180 μm; and 2 μm guard zones. Tissue thickness was determined at each counting field. The coefficient of error was < 0.1.

### Unbiased stereological estimation of dopaminergic neurons in the SN and the VTA

Dopaminergic neuronal number was estimated using unbiased stereology according to the optical fractionator principle described by West et al., [33]. Briefly, the number of tyrosine hydroxylase (TH)-immunoreactive neurons and Nissl^+^ cells were determined by evaluating every fourth coronal Sect. (1/4) covering the entire SN or ventral tegmental area (VTA) structures. The region of interest (SN or VTA) was delineated at low magnification (10X) and then the dopaminergic neurons were counted under an oil immersion objective (40X). TH^+^ neurons were counted in a blinded fashion and the results were expressed as the mean ± standard error of the total number of TH^+^ neurons in each structure. Analysis was performed using the MBF Stereo Investigator software (MBF Bioscience, Williston, VT, USA). The parameters used for the stereological analysis were as follows: grid size, 150 × 150 μm; counting frame, 75 × 75 μm; and 2 μm guard zones. Tissue thickness was determined at each counting field. The coefficient of error was < 0.1.

### Microdissection and protein extraction

The microdissection procedure was performed using cryostat (CM1900, Leica) at -17 °C. The hemispheres stored at -80 °C were cut in coronal Sect. (500 μm thick) with a matrix. The specific structures (motor cortex, striatum, hippocampus, and SN) were recovered on coronal slices with a scalpel, put in pre-weighed tubes and stored at -80 °C until protein extraction.

For protein extraction, after the weight of the tissue was evaluated (weight full tube – weight empty tube), protein extraction solution (1% Sodium Deoxycholate, 1% Triton, 0,1% SDS, 1% Protease /Phosphatase Inhibitor in RIPA Buffer) was added to the tube [Volume = 10 x weight (mg)] on ice. The tissue was homogenized with Pellet Pestle (Z35994-7, Sigma), followed by 3 sessions of bath sonication (2’15 at 90% intensity with 1 s pulse ON then 2 s OFF) (Fisherbrandtm Model 505 sonicator) and vortex-shaking between sessions. After sonication, samples were mixed using a rotator (Benchmark Scientific Roto-Mini Plus R2024) for 30 min at 4 °C, then centrifuged at 13,000 rpm during 30 min at 4 °C. The supernatants were recovered as the soluble fraction, and the pellets were stored for use for the insoluble fraction procedure. The protein concentrations of soluble fraction were evaluated using a BCA kit (PI23225, Thermo Scientific, Waltham, USA).

For the insoluble fraction procedure, pellets were resuspended with 100 µL of protein extraction solution (1% Sodium Deoxycholate, 1% Triton, 0.5% SDS, 1% Protease /Phosphatase Inhibitor in RIPA Buffer), followed by 2 sessions of bath sonication (2’15 at 90% intensity with 1 s pulse ON then 2 s OFF) (Fisherbrandtm Model 505 sonicator) and vortex-shaking between sessions. After sonication, samples were mixed using a rotator (Benchmark Scientific Roto-Mini Plus R2024) for 30 min at 4 °C, then centrifuged at 13,000 rpm during 3 min at 4 °C. The supernatants were recovered as insoluble fraction.

### Western blot (SDS-PAGE)

Soluble fractions were dissolved in 4x Laemmli buffer (161–0747; Bio-Rad, Hercules, CA, USA) and H_2_O milliQ and incubated at 95 °C for 15 min for DNA denaturation. 20 µl of this mix (corresponding to 20–30 µg of proteins) was loaded per well in a 12% SDS-PAGE gel. Gels were run at 180 V for 55 min, and the proteins were transferred to nitrocellulose membranes using the Trans-Blot Turbo™ Transfer system (Bio-Rad, Hercules, CA, USA). The membranes were heated in 0.1 M PBS to increase epitope exposure and incubated with blocking solution (3% gelatin from cold water fish in PBS-Tween 0.1%) at RT for 1 h prior to overnight incubation with primary antibodies in the blocking solution (See Table 1). Membranes were then washed 3 times with PBS-Tween 0.1% (PBS-T) (10 min), incubated with the appropriate secondary antibodies, either 680RD-conjugated or 800 W-conjugated (LI-COR Lincoln, NE, USA) (see Table 1) and finally washed 3 times (10 min) with PBS-T. For the validation of the specificity of phospho-α-syn (pS129) antibodies, after SDS-PAGE and protein transfer, nitrocellulose membrane were blocked with 3% fish-gelatin in TBS with 0.1% Triton X-100 for 1 h at room temperature and then incubated for 10 min at 37 °C in 5 ml of 1x TBS solution containing 500 units of Calf intestinal alkaline phosphatase (CIP) (M0525; New England Biolabs, Massachusetts, United States). Following CIP treatment, membranes were incubated with pS129 antibodies (see Table 1), then washed 3 times with TBS-Tween 0.1% (TBS-T) (10 min) and incubated with the appropriate secondary antibodies. Visualization and quantification were carried out with the LI-COR Odyssey scanner and software (LI-COR Lincoln, NE, USA). Full Western blots are shown in Suppl. Figure 8.

### Dot blot analysis

The accumulation of fibrillar and/or oligomeric forms of α-syn from the insoluble fractions (see Microdissection and protein extraction) from mouse brains were confirmed by dot blot measurements using the dot blot system from (Core LifeScience, Niguel, CA, USA). First, the protein concentrations of pooled insoluble fractions were evaluated using a BCA kit (PI23225, Thermo Scientific, Waltham, USA). Then a 100 ng of the controls (recombinant monomeric α-syn or pre-formed fibrils (PFF)) and 100 ng of protein extracted from control, mTurq, and hα-syn groups were spotted on nitrocellulose membranes. Membranes were allowed to dry prior to incubation with blocking solution (3% gelatin from cold water fish in PBS-Tween 0.1%) at RT. The membranes were then incubated overnight with primary antibodies in the blocking solution (See Table 1). Membranes were then washed 3 times with PBS-Tween 0.1% (PBS-T) (10 min), incubated with the appropriate secondary antibodies, either 680RD-conjugated or 800 W-conjugated (LI-COR Lincoln, NE, USA) (see Table 1) and finally washed 3 times (10 min) with PBS-T. Visualization and quantification were carried out with the LI-COR Odyssey scanner and software (LI-COR Lincoln, NE, USA). At least 3 independent experiments were analyzed for dot blot analysis.

### RT-QuIC (real-time quaking-induced conversion)

RT-QuIC experiments were performed according to previously described protocols [34]. Briefly, samples from the experimental controls included 20 µg/ml of recombinant monomeric α-syn as substrates, and 1.5 µg/ml of purified α-syn PFFs as seeds. Likewise, 1.5 µg/ml of the pooled hα-syn and mTurq samples were used as seeds. A volume of 100 µl of reaction mixtures were pipetted in triplicates in black clear bottom 96-well plates. The reaction mixture was composed of the following: 150 mM NaCl, 1 mM EDTA, 10 µM Th-T, 70 mM SDS, and 20 µg/ml of recombinant monomeric α-syn as substrate for the experiment, in PBS (pH 7.1). Negative controls included monomeric α-syn alone, and α-syn PFFs alone. Plates were covered with sealing tape and incubated in a plate reader (41 °C, with orbital shaking at 425 rpm for 1 min followed by a 2-min rest period). This program was left to run for up to 5 days. The Th-T fluorescence was measured (excitation 435 nm; emission 485 nm) every 30 min using (Bio- Tek Cytation 5 Multi-Mode Readers) plate reader. For RT-QuIC, experiments were performed at least 3 times.

### Filter retardation blotting assay

Samples from the RT-QuIC experiments were diluted in lysis buffer containing 1% SDS and incubated at RT for 10 min. The vacuum manifold (Core LifeScience, Niguel, CA, USA) was prepared by using thin filter paper pre-soaked in water and placed on the manifold. A cellulose acetate membrane (pore size 0.2 µM) (CA022005; SterliTech, Kent, WA, USA) was soaked in PBS containing 1% SDS and placed on top of the filter paper on the manifold. The manifold was tightly closed, and samples were loaded in triplicates into the wells. The samples were then filtered through the membrane by application of a vacuum. After filtration, the membrane was washed 2 times (5 min) with PBS containing 0.1% SDS. After filtration, the membrane was washed 2 times (5 min) with PBS only. The immunoblotting was carried out as described in the SDS-Page protocol. At least three independent experiments were analyzed for retardation assays.

### Statistical analysis

One-way ANOVA followed by Tukey’s multiple comparison tests were used to compare differences between experimental conditions for a single time-point and brain region. Two-way ANOVA followed by Tukey’s multiple comparison tests were used to compare differences between experimental conditions for different brain regions, and differences between regions. Two-way repeated measures ANOVA followed by Tukey’s post hoc tests were used to compare differences between experimental conditions over time, and differences between time-points. One sample t-tests were used for each experimental group to determine whether the percentage of spontaneous alternation obtained in the Y-maze test was above chance level (50%).

All values were expressed as the means ± s.e.m. and the software used for the statistical analysis was Prism v.6 (GraphPad, La Jolla, CA, USA). p < 0.05 was required for rejection of the null hypothesis.

## Results

### Systemic delivery of AAV-PHP.eB results in a global-scale expression of hα-syn in the brain

**Fig. 1:**
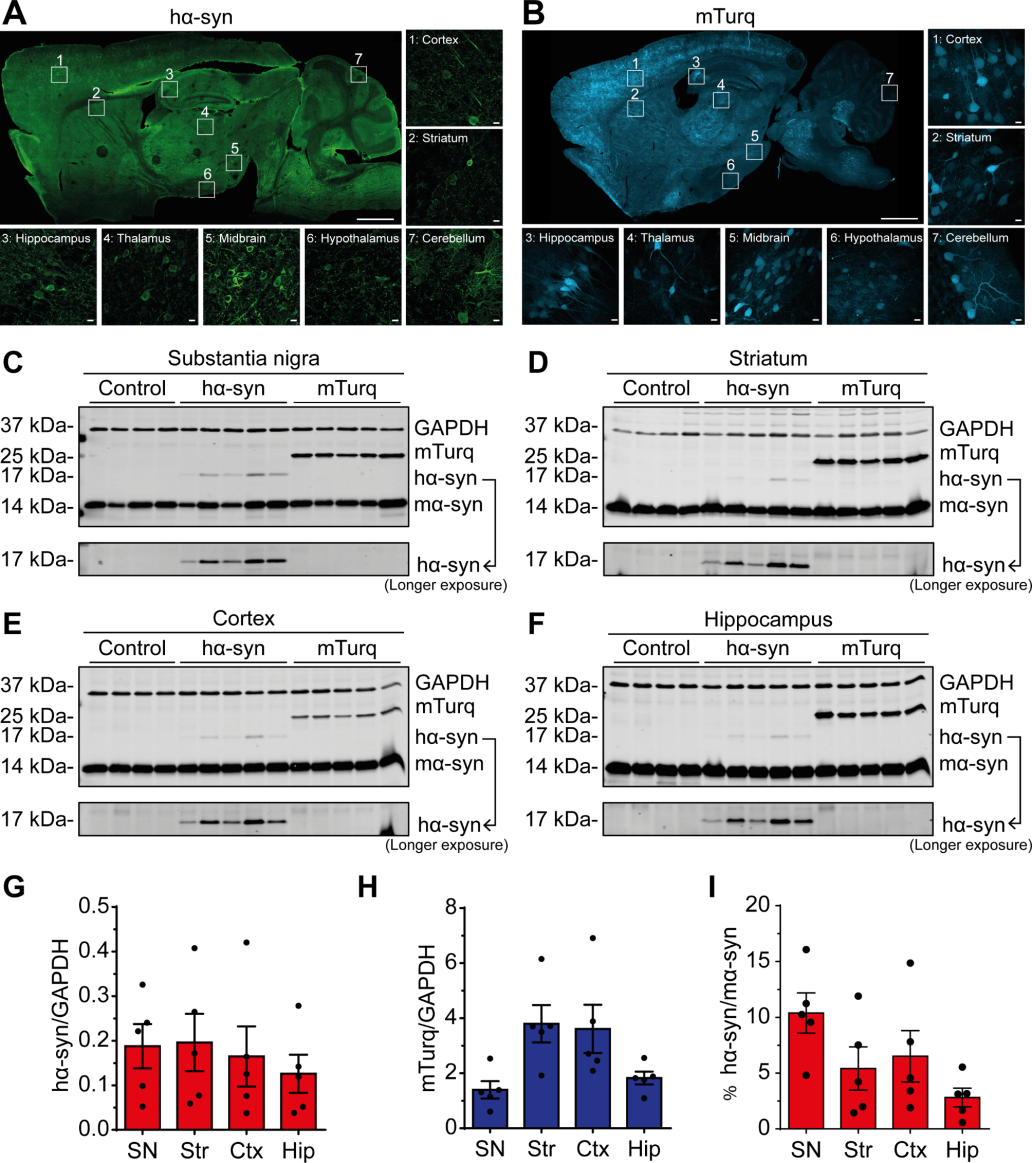
AAV-PHP.eB hα-syn transduces several cerebral regions and induces widespread expression of the transgenes in the brain 2-weeks post-AAV delivery. (**A-B**) Representative confocal microscopy images of sagittal brain slices of mice overexpressing (**A**) hα-syn or (**B**) mTurq (scale bar = 1 mm). Numbered boxes represent zoomed images of the different brain regions: [1] cortex, [2] striatum, [3] hippocampus, [4] thalamus, [5] midbrain, [6] hypothalamus, and [7] cerebellum (scale bar = 10 μm). (**C-F**) Western blot analysis of hα-syn, mα-syn and mTurq protein levels in the soluble protein fraction from the (**C**) substantia nigra, (**D**) striatum, (**E**) motor cortex, and (**F**) hippocampus. GAPDH was used as a loading control. (**G-H**) Quantification of the levels of (**G**) hα-syn and (**H**) mTurq in different brain regions normalized to GAPDH. (**I**) Ratio of hα-syn protein levels compared to endogenous mα-syn. The data are expressed as the means ± s.e.m. (n = 4–5 mice per experimental condition). SN: substantia nigra, Str: striatum, Ctx: cortex, Hip: hippocampus. Full Western blots are shown in Suppl. Figure 8

To induce a large-scale hα-syn overexpression in mice brains, we delivered AAV- PHP.eB viral particles overexpressing hα-syn fused to a myc tag to the blood circulation, via intravenous retro-orbital injection, to 3-month-old C57Bl/6 mice. The addition of the myc tag helps with enhancing the distinction between the endogenous and overexpressed α-syn, with minimal impact on the protein’s structure and behavior [35, 36]. As control groups, we injected animals with viral particles overexpressing the fluorescent protein mTurquoise2 (mTurq) or injected with PBS (control). First, we performed post-mortem tissue analysis, 2-weeks post-injection, to evaluate the transduction efficiency and to assess transgene expression levels in different brain regions. Using anti-hα-syn antibody (LB509), we observed that exogenous hα-syn signal was widely detected in all brain regions, with a marked staining in the neuronal soma and neurites in the cortex, the striatum, the hippocampus, the thalamus, the midbrain, and the hypothalamus, as well as the cerebellum (Fig. 1A). Interestingly, we also observed an abundant hα-syn punctate staining, consistent with a presynaptic localization of α-syn in different brain regions [13] (Fig. 1A). Of note, the absence of signal using LB509 antibody in the control group confirmed the specificity of α-syn staining in hα-syn-injected group (Suppl. Figure 1A). Moreover, mTurq signal was also detected in the soma and cell neurites in all brain regions, as previously reported [22] (Fig. 1B). It is important to mention that, although the systemic injection of AAV particles was unilateral, the expression of the two transgenes was detected bilaterally with a similar intensity in the two hemispheres, thus representing an important advantage for the use of the present delivery approach (Suppl. Figure 1B-D).

It is important to highlight that despite the well-known strong neuronal tropism of AAV-PHP.eB [22, 37, 38], there was notable variability in the quantification of NeuN^+^ cells expressing the transgenes, depending on the specific brain region examined. While certain regions showed a lower to moderate percentage of transgene-expressing neurons (e.g., 15% in the striatum, 17% in the hippocampus, and 26% in the cortex), the SN displayed a significantly higher rate of neuronal transduction (48%) at 2-weeks post-injection (Suppl. Figure 2 A). As previously reported [22], we also observed a minor expression of the transgenes in other brain cell types, including astrocytes and microglia (Suppl. Figure 2B and C).

To further assess the transgene expression levels in different brain regions, we extracted the soluble protein fraction from the substantia nigra (SN), the striatum, the cortex and the hippocampus and performed Western blot analysis. Using an anti-α-syn antibody (Syn1/BD Lab), we detected a band at 17 kDa corresponding to the exogenous hα-syn fused to the myc tag (Fig. 1C-F). Analysis of this band intensity revealed a disparity in the transgene expression levels between animals in each condition, probably due to the intravenous retro-orbital injection efficiency (Fig. 1G **and H**). Moreover, the expression levels of hα-syn and mTurq were also different between the brain regions, (Fig. 1G **and H**). As anti-α-syn antibody (Syn1/BD Lab) also recognizes endogenous mouse α-syn (mα-syn), we were able to compare exogenous and endogenous α-syn levels, and quantification estimated that the hα-syn levels is representing between 3 and 10% of the mα-syn (Fig. 1I). Finally, we evaluated the stability of the transgenes expression levels and observed a consistent hα-syn and mTurq signals in the different brain regions, 3-months post-injection (Suppl. Figure 3 A and B). Interestingly, the levels of hα-syn, in comparison to its endogenous counterpart, exhibited a progressive increase over time across all brain regions, ultimately reaching approximately 20–30% of the endogenous α-syn level at 3-months post-injection (Suppl. Figure 3 C-I). Collectively, these results demonstrate that systemic delivery of AAV-PHP.eB particles resulted in discrete, but widespread, stable, and bilateral hα-syn overexpression in the mouse brain.

### Systemic delivery of hα-syn induced progressive motor impairment

**Fig. 2:**
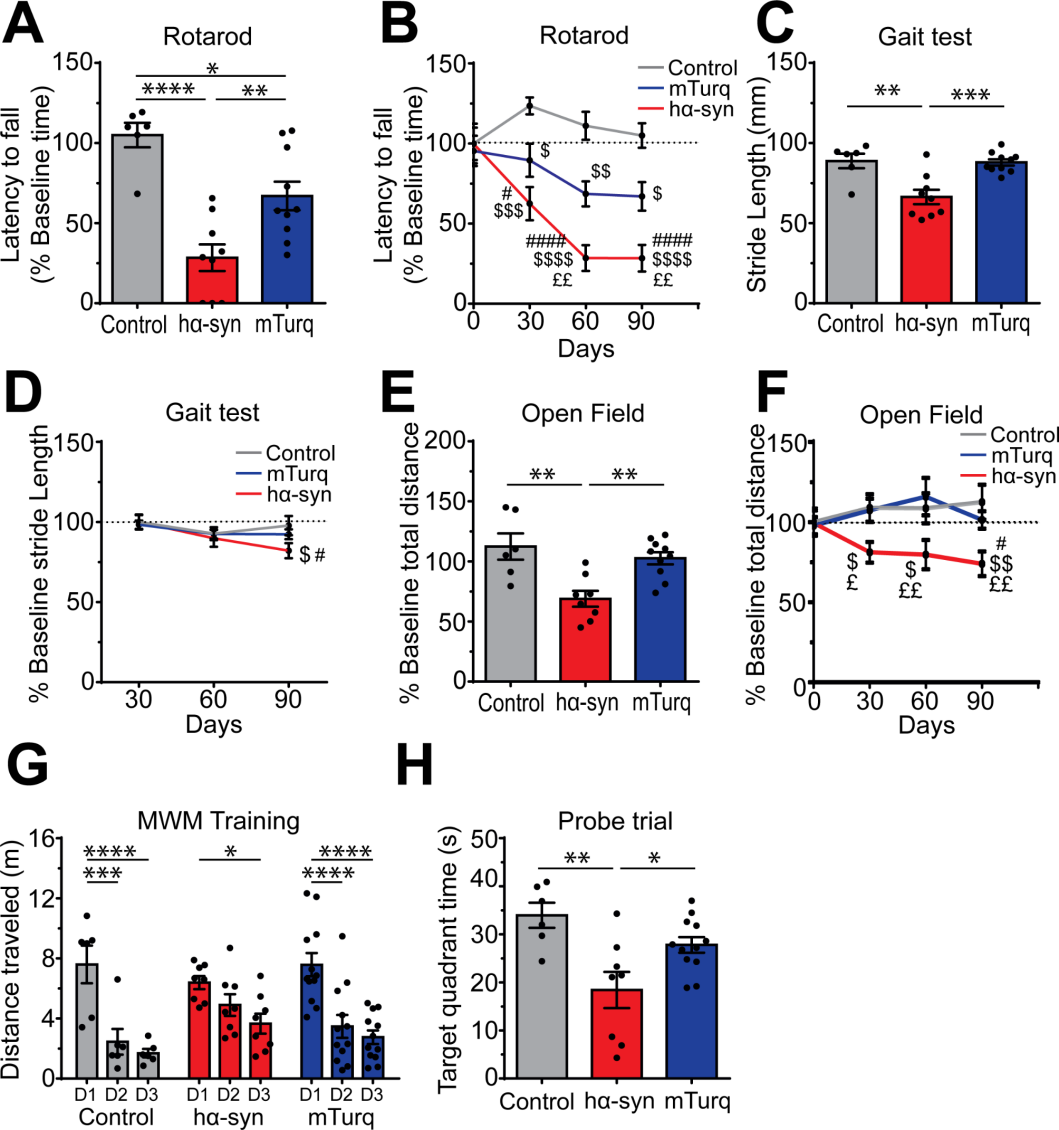
Overexpression of hα-syn induces motor and cognitive impairment. (**A**) Evaluation of motor impairment using the rotarod test 3-months post-AAV delivery. (**B**) Progression of animals’ performances on the rotarod test, evaluated at 1-month, 2-months, and 3-months post-AAV delivery. (**C**) Evaluation of the animals’ motor coordination using the gait test 3-months post-AAV delivery. (**D**) Progression of animals’ deficits on the gait test, evaluated at 1-month, 2-months, and 3-months post-AAV-delivery. (**E**) Evaluation of animals’ performance in the open field at 3- months post-AAV-delivery. (**F**) Progression of animals’ performances in the open field, evaluated at 1-month, 2-months, and 3-months post-AAV delivery. (**G**) Evaluation of animals’ performance in the training phase of the MWM test at 3-months post-AAV-delivery. (**H**) Evaluation of animals’ performance in the probe trial of the MWM test at 3-months post-AAV-delivery. The data are presented as the means ± s.e.m. (n = 6–10 mice per experimental condition). One-way ANOVA followed by Tukey’s multiple comparisons test; (**A**, **C**, **E** & **H**) * p ≤ 0.05, ** p ≤ 0.01, *** p ≤ 0.01 and **** p ≤ 0.0001. Two-way repeated measures ANOVA followed by Tukey’s multiple comparisons test; (**B**, **D** & **F**) $ p ≤ 0.05, $$ p ≤ 0.01, $$$ p ≤ 0.001 and $$$$ p ≤ 0.0001 versus control group; £ p ≤ 0.05 and ££ p ≤ 0.01 versus mTurq group; # p ≤ 0.05 versus baseline for each group; (**G**) * p ≤ 0.05, ** p ≤ 0.01, *** p ≤ 0.01 and **** p ≤ 0.0001. MWM: Morris water maze

To investigate if the large-scale hα-syn overexpression in mouse brains was associated with the manifestation of behavioral phenotype, we performed a battery of tests to evaluate the animals’ motor performance. Three months post-viral delivery, we analyzed animals’ motor coordination, endurance, and balance using the rotarod test. Results revealed that mice overexpressing hα-syn exhibited a significant short latency to fall, compared to the control and mTurq groups [F [2, 22] = 17.07, p < 0.0001; hα-syn vs. control: p < 0.0001; hα-syn vs. mTurq: p = 0.0079], suggesting that hα-syn overexpression affected animals’ motor coordination (Fig. 2A). Of note, the rotarod test analysis overtime revealed that hα-syn overexpression affected animal performance as early as 1-month post-viral delivery and this deleterious effect worsened with time and reached a plateau at 2 months [effect of time: F [3, 66] = 7.711, p = 0.0002; 1 month vs. baseline: p = 0.0299; 2 months vs. baseline: p < 0.0001; 3 months vs. baseline: p < 0.0001] (Fig. 2B). At 3-months post-injection, the mTurq group exhibited a moderated decline in the animals’ performance in the rotarod test, compared to the control group [mTurq vs. control: p = 0.0201] (Fig. 2A). However, this impairment did not reach statistical significance when compared to the animals’ baseline performance [1 month vs. baseline: p = 0.9661; 2 months vs. baseline: p = 0.1493; 3 months vs. baseline: p = 0.1140] (Fig. 2B). This observation suggests that mTurq overexpression had no significant impact on the animals’ overall performance, and the observed effect at 3-months is likely attributed to fluctuations in the performance of the control group. Moreover, analysis of movement coordination, using the gait test, revealed significant deficits in mice overexpressing hα-syn at 3-months post-viral delivery, compared to the control group [F [2, 22] = 12.62, p = 0.0002; hα-syn vs. control: p = 0.0014], whereas no effect was observed in the mTurq group [mTurq vs. control: p = 0.9855] (Fig. 2C). This gait abnormality appears progressively and reaches significant levels after 3-months post-viral delivery [effect of time: F [2, 44] = 2.834, p = 0.0696; 2 months vs. 1 month: p = 0.4724; 3 months vs. 1 month: p = 0.0132] (Fig. 2D). Furthermore, hα-syn overexpression induced impairment of mice locomotor activity at 3-months post-viral delivery, as evaluated by the open field test, compared to the control and mTurq groups [F [2, 21] = 9.845, p = 0.0010; hα-syn vs. control: p = 0.0016; hα-syn vs. mTurq: p = 0.0049] (Fig. 2E). This deficit appears very early after hα-syn overexpression (1-monthpost-viral delivery) [effect of group: F [2, 21] = 7.908, p = 0.0028; hα-syn vs. control: p = 0.0465; hα-syn vs. mTurq: p = 0.0355] and progresses overtime [effect of time: F [3, 63] = 0.3033; p = 0.8229; hα-syn, 1 month vs. baseline: p = 0.1929; 2 months vs. baseline: p = 0.1315; 3 months vs. baseline: p = 0.0193] (Fig. 2F). Finally, no effect was observed after analysis of the cylinder test [F [2, 22] = 0.7333, p = 0.4917] and the grip force test F [2, 22] = 1.620, p = 0.2207] (Suppl. Figure 4 A and B).

Given the fact that non-motor symptoms are also a major characteristic of PD, we investigated the impact of hα-syn overexpression on mice cognitive performance, 3- months post-viral delivery. Our data revealed deficits in learning abilities in mice overexpressing hα-syn, compared to the control or mTurq groups during the training of the Morris water maze (MWM) test (Fig. 2G). This learning deficit was significant with the probe trial [F [2, 23] = 7.468, p = 0.0032; hα-syn vs. control: p = 0.0028; hα-syn vs. mTurq: p = 0.0342] (Fig. 2H). Finally, no effect of hα-syn overexpression was observed on the animals’ spatial working memory and anxiety as assessed using the Y-maze [F [2, 24] = 1.338, p = 0.2813] and elevated-plus maze (EPM) [F [2, 26] = 0.3687, p = 0.6952] tests, respectively (Suppl. Figure 4 C and D). Collectively, our results demonstrate that widespread hα-syn overexpression in the brain was associated with early onset and progressive motor impairment and learning deficits.

### Widespread hα-syn overexpression in the mouse brain induced selective dopaminergic neuronal loss in the midbrain

We next evaluated if the wide-scale overexpression of hα-syn could induce neuronal degeneration in the brain. As exogenous transgenes were equally expressed in both brain hemispheres (Suppl. Figure 1B-D), we post-fixed one hemisphere in paraformaldehyde (PFA) for the immunohistochemistry and the other hemisphere was used for the collection of soluble and insoluble protein fractions for biochemical analysis.

**Fig. 3:**
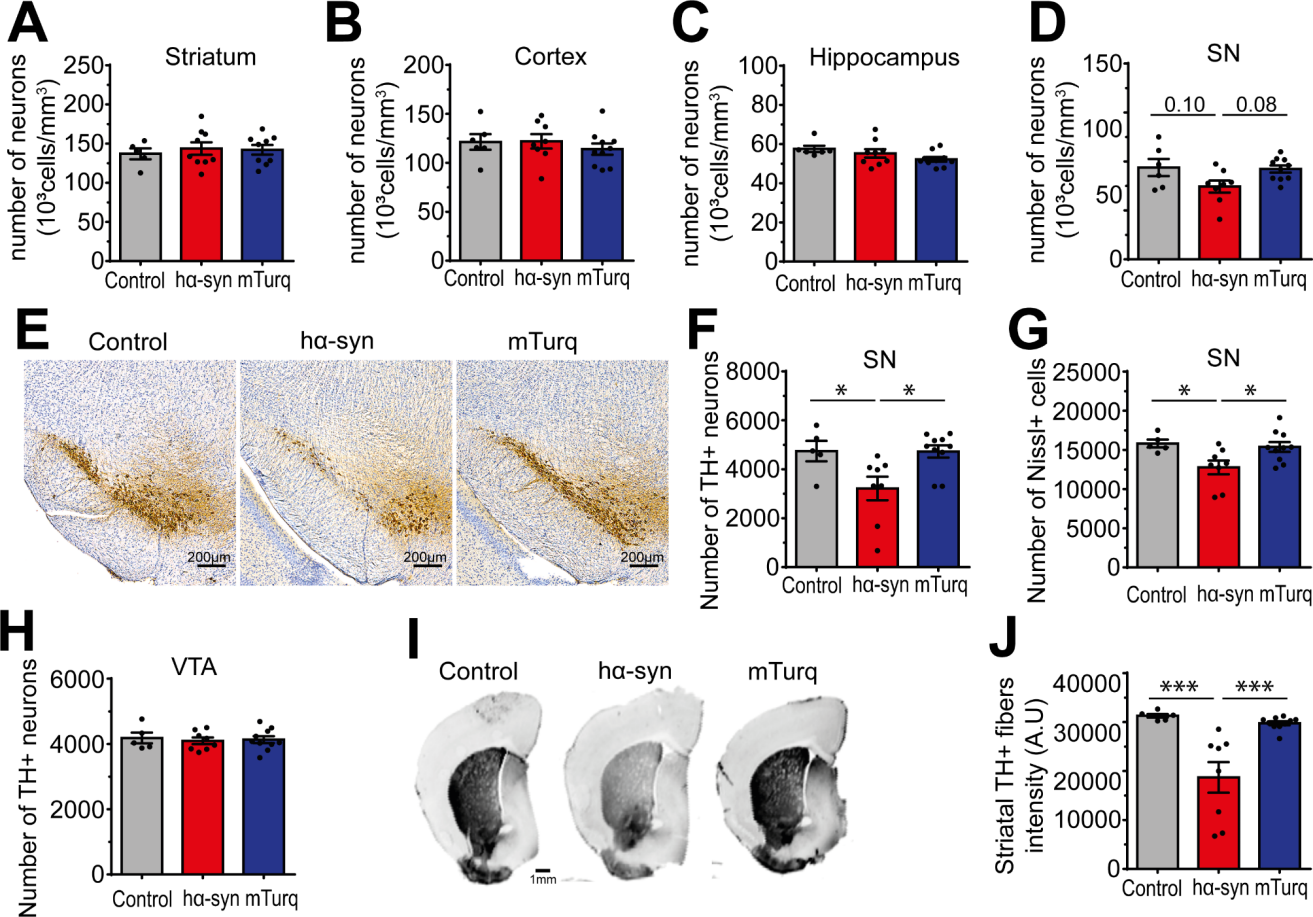
Widespread overexpression of hα-syn induces selective dopaminergic neuronal loss in substantia nigra 3-months post-AAV delivery. (**A-D**) Stereological quantification of NeuN^+^ cells in the (**A**) striatum, (**B**) motor cortex, (**C**) hippocampus, and (**D**) SN. (**E**) Representative bright-field microscopy images of coronal brain slices illustrating TH^+^ neurons in the midbrain. Tissue was counterstained with Nissl stain (scale bar = 200 μm). (**F-G**) Stereological quantification of (**F**) TH^+^ dopaminergic neurons and (**G**) Nissl^+^ cells) in the SN. (**H**) Stereological quantification of TH^+^ dopaminergic neurons in the VTA. (**I**) Representative images of coronal brain slices illustrating the TH staining in the striatum (scale bar = 1 mm). (**J**) Quantification of TH staining optical density values in the striatum. The data are expressed as the means ± s.e.m. (n = 6–10 mice per experimental condition). One-way ANOVA followed by Tukey’s multiple comparisons test; * p ≤ 0.05, *** p ≤ 0.001. SN: substantia nigra; TH: tyrosine hydroxylase; VTA: ventral tegmental area

First, we evaluated the neuronal density (NeuN^+^ cells/mm^3^) in different brain regions, 3-months post-viral delivery. Analysis showed that neither hα-syn nor mTurq overexpression affected the total number of neuronal cells in the striatum [F [2, 20] = 0.1798, p = 0.8368], the cortex [F [2, 21] = 0.4501, p = 0.6436],the hippocampus [F [2, 22] = 2.313, p = 0.1225] or the SN [F [2, 21] = 3.393, p = 0.0529] (Fig. 3A-D). Then, we focused our analysis on the dopaminergic neurons, the principal neuronal population of the SN affected in PD [39–43]. Using a stereological approach for the quantification of the total number of TH^+^ neurons in this region, we observed a significant reduction in the number of dopaminergic neurons in the hα-syn group, when compared to the control or mTurq groups [F [2, 20] = 5.447; p = 0.0129; hα-syn vs. control: p = 0.0477; hα-syn vs. mTurq: p = 0.0172] (Fig. 3E **and F**). Moreover, the reduction of the number of Nissl^+^ cells in the SN in the hα-syn group confirmed that TH^+^ neuronal loss was indeed attributed to neurodegeneration rather than a loss of the dopaminergic phenotype [F [2, 20] = 4.805, p = 0.0198; hα-syn vs. control: p = 0.0420; hα-syn vs. mTurq: p = 0.0363] (Fig. 3G). Interestingly, stereological quantification of the TH^+^ neurons in the VTA showed that the dopaminergic neurons were preserved in this region, despite hα-syn overexpression [F [2, 20] = 0.1125, p = 0.8942; hα-syn vs. control: p = 0.8846; hα-syn vs. mTurq: p = 0.9672] (Fig. 3H), thus confirming the selective vulnerability of the dopaminergic neurons of the SN and the resistance of VTA dopaminergic neurons to α-syn-induced toxicity [44–46]. Finally, analysis of the integrity of the nigrostriatal pathway showed a significant decrease in the dopaminergic fiber density in the striatum in the hα-syn overexpression group, compared to the control and mTurq groups [F [2, 21] = 13.63, p = 0.0002; hα-syn vs. control: p = 0.0006; hα-syn vs. mTurq: p = 0.0005], confirming the degeneration of the dopaminergic nigral terminals associated with the neuronal loss in the SN (Fig. 3I **and J**). Of note, degeneration of the nigrostriatal pathway was associated to a synaptic dysfunction specifically in the striatum, as reflected by the significant decrease of synaptic markers (drebrin [F [2, 11] = 6.354, p = 0.0146; hα-syn vs. control: p = 0.0338; hα-syn vs. mTurq: p = 0.0223], PSD95 [F [2, 11] = 4.023, p = 0.0488; hα-syn vs. control: p = 0.0489; hα-syn vs. mTurq: p = 0.1567] and synaptophysin [F [2, 11] = 10.22, p = 0.0031; hα-syn vs. control: p = 0.0129; hα-syn vs. mTurq: p = 0.0040]) in the hα-syn overexpressing mice, compared to the control and mTurq groups (Suppl. Figure 5), thus mimicking the early neuropathological events observed in the brain of PD patients [47–50]. Altogether, these observations demonstrate that, despite the widespread overexpression in the brain, hα-syn accumulation induced a selective loss of the dopaminergic neurons in the midbrain, mimicking the neurodegeneration observed in PD. Moreover, compared to current α-syn-based animal models, our model presents a suitable tool to investigate selective dopaminergic neuronal vulnerability in PD.

### Dopaminergic neuronal loss in the substantia nigra is associated with the accumulation of pathological forms of α-syn

To investigate if the widespread overexpression of hα-syn and the dopaminergic neuronal loss at 3-months post-viral delivery are associated to abnormal accumulation of pathological α-syn, first, we verified the presence of the disease-associated phosphorylated form of α-syn at the residue Ser129 (pS129) in different brain regions. Unexpectedly, immunohistochemistry did not reveal any accumulation of pS129 α-syn nor the formation of dystrophic Lewy neurites in the cortex, the striatum, the hippocampus, or the midbrain (Suppl. Figure 6 A). This observation suggests that ⍺-syn pathology is either absent or undetectable due to the low levels of the exogenous overexpressed protein.

**Fig. 4:**
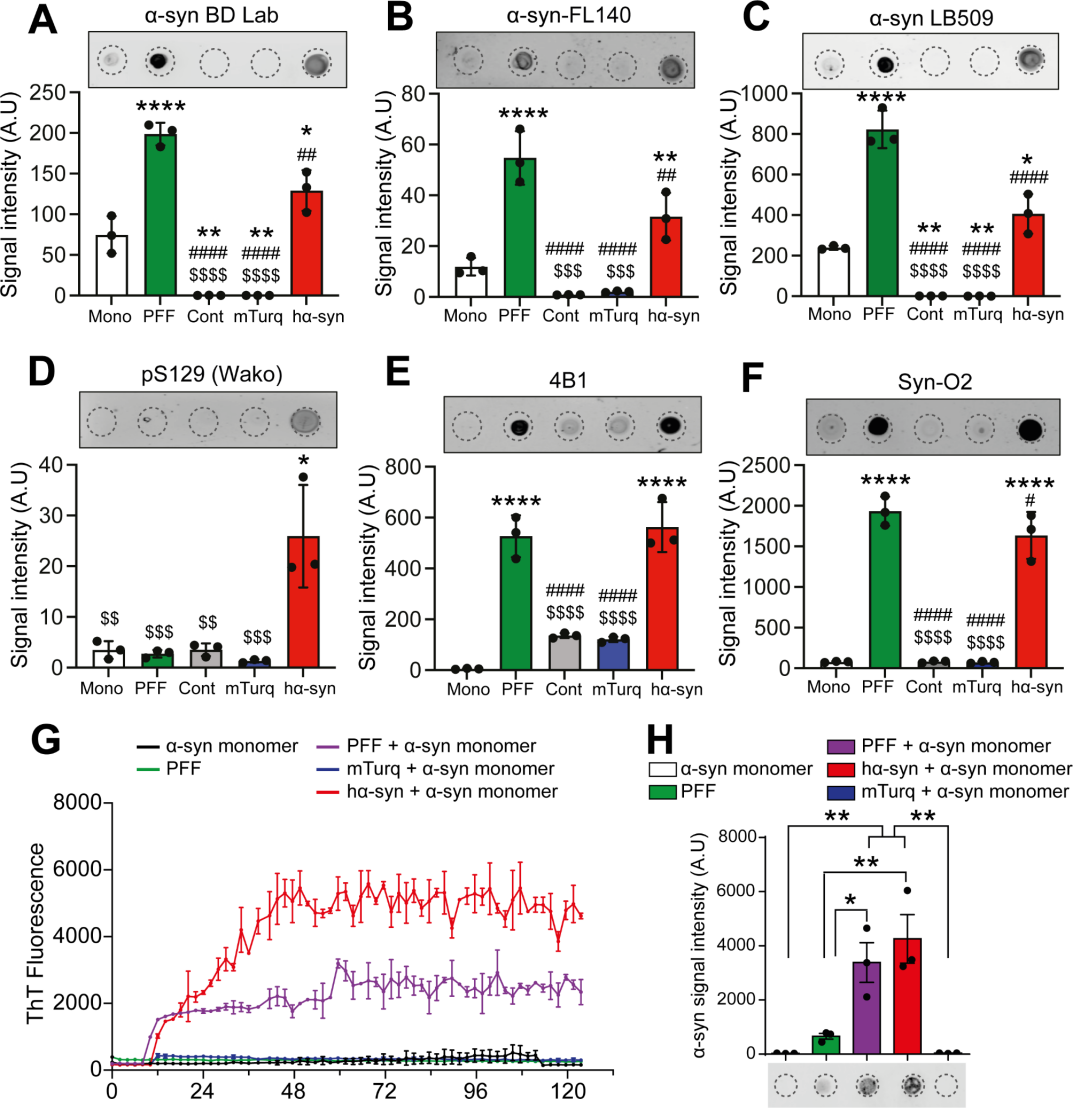
Overexpression of hα-syn induces the formation of α-syn aggregates in the substantia nigra 3-months post-AAV delivery. (**A-E**) Dot blot analysis and quantification of 100 ng of protein prepared from the insoluble fraction of the SN of control, mTurq, and hα-syn mice (n = 6 mice per experimental condition). Recombinant monomeric α-syn (Mono) and PFF were used as controls. Membranes were evaluated for protein expression using the following antibodies: (**A**) Syn1/BD Lab, (**B**) α-syn-FL140, (**C**) α-syn LB509, (**D**) pS129 (Wako), (**E**) 4B1, and (**F**) Syn-O2. The data are presented as the means ± s.e.m. (n = 3). One-way ANOVA followed by Tukey’s multiple comparisons test; * p ≤ 0.05, ** p ≤ 0.01, **** p ≤ 0.0001 versus Mono; ## p ≤ 0.01, #### p ≤ 0.0001 versus PFF; $$$ p ≤ 0.001, $$$$ p ≤ 0.0001 versus hα-syn. (**G**) Representative RT-QuIC analysis illustrating the kinetics of recombinant α-syn aggregation in the presence of protein from the insoluble fraction of the SN of mTurq, and hα-syn mice (n = 6 mice per experimental condition). Monomeric α-syn (Mono) and PFF were used as controls. The average ThT fluorescence intensity was plotted against time. (**H**) Filter retardation assay and quantification of α-syn protein levels (α-syn-FL140) showing the accumulation of α-syn aggregates in the insoluble fractions of hα-syn and recombinant α-syn PFFs after RT-QuIC. The data are presented as the means ± s.e.m. (n = 3). One-way ANOVA followed by Tukey’s multiple comparisons test; * p ≤ 0.05, ** p ≤ 0.01. Mono: α-syn monomer; PFF: pre-formed fibrils; RT-QuIC, real-time quaking-induced conversion; ThT, thioflavin T. Antibody detection: Syn1/BD Lab and α-syn-FL140: mouse and human α-syn; α-syn LB509: human α-syn; pS129: phosphorylated α-syn at S129; 4B1: Non pS129 α-syn aggregates; Syn-O2: α-syn aggregates. Full dot blots and filter retardation membranes are shown in Suppl. Figure 8

Then, we focused our analysis on the SN, and performed dot blot analysis of protein extracts from the insoluble fraction from the midbrain, using a battery of antibodies raised against monomeric α-syn, namely Syn1/BD Lab, α-syn-FL140 and α-syn LB509. Significant levels of α-syn expression were observed using three different antibodies in the protein extracts from mouse brains overexpressing hα-syn, as well as in the positive control samples of human recombinant α-syn (Mono and PFF α-syn) (Syn1/BD Lab [F [4, 10] = 81.41, p < 0.0001], α-syn-FL140 [F [4, 10] = 58.40, p < 0.0001] and α-syn LB509 [F [4, 10] = 96.08, p < 0.0001]). However, no α-syn signal was detected in the control or the mTurq samples, indicating that only the exogenous hα-syn, and not the endogenous mα-syn, was present in the insoluble protein fraction (Fig. 4A-C). We also examined pS129 levels and we observed a weak signal in hα-syn mice protein extracts (pS129-Wako [F [4, 10] = 15.10, p = 0.0003], pS129-Abcam [F [4, 10] = 12.39, p = 0.0007], and pS129-Ghanem et al. 2022 [F [4, 10] = 111.7, p < 0.0001]), while no staining was observed in the control and mTurq groups, as well as in the recombinant proteins (Fig. 4D and Suppl. Figure 6B and C). The specificity of the used pS129 antibodies and the expected size of the phosphorylated α-syn were further confirmed by western blot (Suppl. Figure 7) Moreover, we used an antibody, referred to as 4B1, known to specifically recognize β-sheet-rich fibrillar and oligomeric forms of non-phosphorylated S129–α-syn [51], and our data showed a significant accumulation of pathological α-syn in hα-syn mice protein extracts and PFF samples, while a weaker signal was observed in the recombinant monomeric protein, the control, and the mTurq groups ([F [4, 10] = 77.12, p < 0.0001]) (Fig. 4E). Moreover, using another antibody specifically raised against oligomeric forms of α-syn, namely Syn-O2 [52], we also detected a high signal intensity in hα-syn mice protein extracts and in the positive control (PFF), but not in the control and the mTurq groups ([F [4, 10] = 191.1, p < 0.0001]) (Fig. 4F). This observation was confirmed using a battery of oligomer-specific antibodies (Syn-O1 [F [4, 10] = 157.4, p < 0.0001], Syn-O3 [F [4, 10] = 142.5, p < 0.0001], Syn-F1 [F [4, 10] = 324.3, p < 0.0001] and Syn-F2 [F [4, 10] = 64.94, p < 0.0001]) (Patent no. EP2961774B1) (Suppl. Figure 6D-G).

Furthermore, using real-time quaking-induced conversion (RT-QuIC), an assay commonly used for ultrasensitive detection of oligomeric and aggregated forms of α-syn monitored by the fluorescence signal emitted by thioflavin T (ThT), we observed an increase in ThT signal in the insoluble fraction extracted from the SN of hα-syn mice, as well as in the positive control (recombinant PFF + α-syn monomers), reflecting the presence of pathological hα-syn forms in the SN (Fig. 4G). No significant ThT signal was detected in the insoluble fractions extracted from the mTurq group, nor in the negative controls (recombinant α-syn monomers or PFF alone) (Fig. 4G). Finally, using a filter retardation assay, we validated that the increase in ThT signal in the insoluble fraction from hα-syn mice and α-syn PFFs, observed at the latest timepoint of the RT-QuIC, is indeed reflecting an enhanced accumulation of aggregated α-syn ([F [4, 10] = 14.82, p = 0.0003]) (Fig. 4H). Altogether, our data demonstrate that, hα-syn overexpression induces the formation of discrete pathological hα-syn oligomers in the midbrain, thus mimicking a cardinal hallmark of PD neuropathology.

### Dopaminergic neuronal loss is associated with neuroinflammatory response in the substantia nigra

**Fig. 5:**
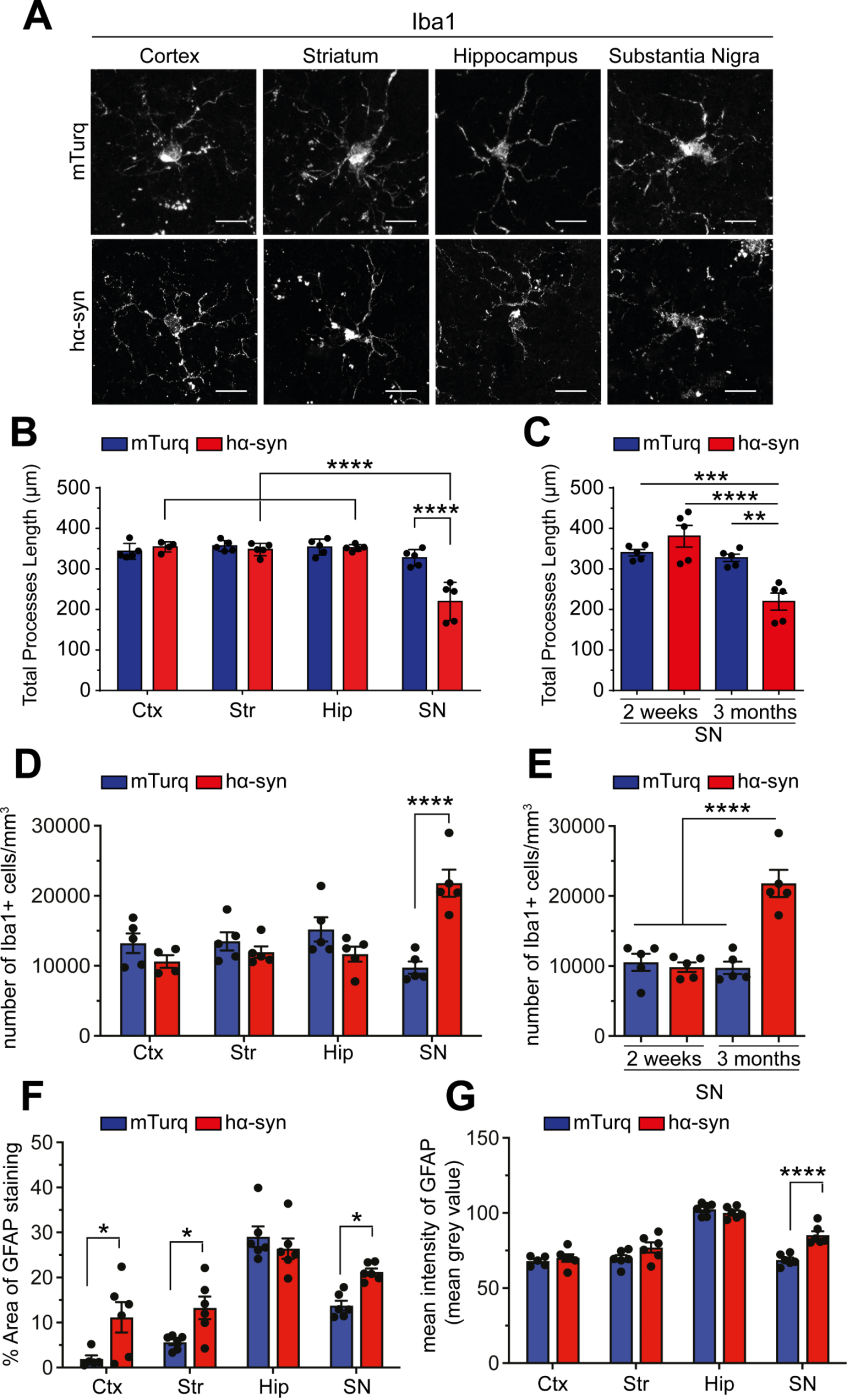
Overexpression of hα-syn induces a neuroinflammatory response in the midbrain. (**A**) Representative fluorescence confocal microscopy images of coronal brain slices illustrating Iba1 staining in different brain regions (cortex, striatum, hippocampus, and SN) (scale bar = 20 μm). (**B**) Quantification of total processes length of microglia in different brain regions of mice overexpressing mTurq or hα-syn, 3-months post-AAV delivery. (**C**) Quantification of total processes length of microglia in the SN of mice overexpressing mTurq or hα-syn, 2-weeks and 3-months post-AAV delivery. (**D**) Quantification of the number of Iba1^+^ cells in different brain regions of mice overexpressing mTurq or hα-syn, 3-months post-AAV delivery. (**E**) Quantification of the number of Iba1^+^ cells in the SN of mice overexpressing mTurq or hα-syn, 2-weeks and 3-months post-AAV delivery. (**F**) Quantification of the percentage area of GFAP staining in different brain regions of mice overexpressing mTurq or hα-syn, 3-months post-AAV delivery. (**G**) Quantification of the mean intensity of GFAP signal/staining in different brain regions of mice overexpressing mTurq or hα-syn, 3-months post-AAV delivery. The data are presented as the means ± s.e.m. (n = 5–6 mice per experimental condition). (**B, D, G & F**) Two-way ANOVA followed by Tukey’s multiple comparisons test; * p ≤ 0.01, *** p ≤ 0.001, **** p ≤ 0.0001. (**C & E**) One-way ANOVA followed by Tukey’s multiple comparison test; ** p ≤ 0.01; *** p ≤ 0.001, **** p ≤ 0.0001. Ctx: cortex; Str: striatum; Hip: hippocampus; SN: substantia nigra

A growing body of evidence indicates that α-syn-induced toxicity is commonly associated with a neuroinflammatory process characterized by the activation of microglia (microgliosis) [53–55] and reactive astrocytes (astrogliosis) in the SN [56, 57]. To investigate if such inflammatory reaction was induced by the systemic hα-syn overexpression in the mouse SN, we assessed microglia activation by quantifying the morphological shift from small soma with ramified processes to the morphology of amoeboid cells with shorter and fewer processes [58, 59]. Analysis of Iba1^+^ microglia morphology in different brain regions, 3-months post-injection, showed a significant and selective decrease of the total processes’ length in hα-syn mouse midbrain [effect of brain region: F [3, 31] = 29.16, p < 0.0001; effect of group: F [1, 31] = 13.59, p = 0.0009; hα-syn vs. mTurq, SN: p < 0.0001], while no such effect was observed in the other brain regions (cortex, striatum, and hippocampus), nor in mTurq mouse brains [hα-syn vs. mTurq, cortex: p > 0.9999; striatum: p > 0.9999; hippocampus: p > 0.9999] (Fig. 5A **and B**). It is important to mention that the neuroinflammatory reaction appears to be progressive and to exacerbate with time, as the effect of hα-syn overexpression on Iba1 morphology in the SN was only observed at 3-months post-injection and not at earlier time points (2-weeks post-injection) ([F [3, 16] = 14.46, p < 0.0001; 3 months vs. 2 weeks: p < 0.0001]) (Fig. 5C). Moreover, hα-syn-induced changes of the microglia morphology was also associated with microgliosis in the SN, reflected by an increase Iba1^+^ cell density, at 3- months post-injection [(Fig. 5D) effect of group: F [1, 31] = 1.343, p = 0.2553; hα-syn vs. mTurq: p < 0.0001 and (Fig. 5E) F [3, 16] = 21.37, p < 0.0001; hα-syn vs. mTurq: p < 0.0001] (Fig. 5D **and E**).

Subsequently, we conducted an examination of potential astrogliosis by evaluating the reactivity of glial fibrillary acidic protein (GFAP) in various brain regions. Consistent with the findings of increased Iba1 reactivity, we noted an elevated proportion of GFAP-immunoreactive area [effect of group: F [1, 39] = 14.62, p = 0.0005; hα-syn vs. mTurq: p = 0.0450] and intensified GFAP immunostaining in the SN overexpressing hα-syn [effect of group: F [1, 39] = 13.72, p = 0.0007; hα-syn vs. mTurq: p < 0.0001] (Fig. 5F **and G**). Interestingly, in contrast to the microgliosis specific to SN, we observed increased GFAP immunoreactivity in all brain regions, except for the hippocampus [hα-syn vs. mTurq, cortex: p = 0.0123; striatum: p = 0.0383; hippocampus: p = 0.8240], suggesting a more widespread astrogliosis in response to hα-syn overexpression (Fig. 5F **and G**). Collectively, these findings indicate that, at 3-month post-injection, the overexpression of hα-syn leads to reactive gliosis and astrogliosis, particularly in the SN, which aligns with the loss of dopaminergic neurons and the buildup of pathological α-syn.

## Discussion

Animal models, mimicking important aspects for human disorders, represent an indispensable tool to decorticate cellular and molecular bases of these diseases and to test new emerging therapeutic strategies. In the present study we report on the creation of a new rodent model of PD, after a unique and non-invasive delivery of AAV-PHP.eB viral particles overexpressing hα-syn in the blood circulation. This approach led to a discrete, but widespread, overexpression of hα-syn in the CNS, with the manifestation of key PD neuropathological features, including selective dopaminergic neuronal loss, PD-like motor and learning deficits, accumulation of pathologic α-syn, and the induction of neuroinflammatory reaction. This model, representing a conciliation between the acute overexpression of viral-based model and the widespread transgene expression of Tg models, provides an accessible and valuable tool to study the role of α-syn in PD pathogenesis, and for the development of effective disease-modifying therapies for PD and related α-synucleinopathies.

### Advantages of using AAV-PHP.eB for the creation of a rodent model of PD

The first advantage of using AAV-PHP.eB is the capacity of these viral particles to cross the BBB and to reach different brain regions [22]. This propriety permits to bypass the use of stereotaxic injection, a technique commonly used for intracerebral viral delivery that presents with several limitations, notably the temporal and spatial restriction of transgene expression and the requirement of specific expertise and equipment [21], thus making this approach accessible and easily implemented by a large number of research groups.

The second major advantage for the use of AAV-PHP.eB, is that a single non-invasive administration of the viral particles resulted in the widespread and stable expression of hα-syn in the brain. Moreover, while the injection of the viral particles was unilateral, the expression of the transgenes was detected bilaterally and virtually affected all the brain regions, thus mimicking the widespread overexpression pattern observed in Tg mice [60], but without the limitations of this approach, notably the high-cost, the lengthy protocol and the elevated number of animals needed [61]. Of note, immunohistochemistry revealed the presence of hα-syn in neuronal cell bodies, as well as in synaptic terminals (puncta signal), thus demonstrating that α-syn was correctly addressed and localized at the physiological neuronal compartments [13].

It is important to acknowledge that, despite its advantages, our model requires some optimization, specifically in reducing intragroup variability. This variability, as reported in numerous previous studies [22, 38], may stem from various factors associated with retro-orbital injections. These factors include needle placement, and injection volume and speed, as well as environmental conditions such as temperature, humidity, and stress levels, all of which can impact the animal’s physiology and vasculature [37]. To enhance the consistency and reliability of retro-orbital viral injections, it is crucial to standardize the injection technique, optimize parameters such as injection volume and speed, and implement rigorous experimental controls.

### Widespread hα-syn overexpression induced progressive motor impairment and selective dopaminergic loss

Analysis of the animals’ behavioral performances revealed a progressive manifestation of motor impairment, specifically the loss of movement coordination (rotarod and gait test), 3-months post-AAV delivery. Interestingly, deficits on the rotarod and in animals’ activity appeared very early (1-month post-injection) and continued to aggravate with time. These results represent an advantage of our new model when compared with α-syn Tg models, in which studies have reported motor deficits in aged animals (8 to 12 months of age) and inconstancies in animals’ behavioral performances [60, 62–66]. It is important to mention that in our model, we did not observe any effect on the cylinder test. This test is commonly used to detect motor deficits in unilateral intracerebral injection of AAV-α-syn [18, 25, 26], supporting that in our model the cellular impact of hα-syn overexpression was equally affecting the two hemispheres.

At the cellular level, post-mortem analysis showed that the large-scale overexpression of hα-syn in the brain resulted in a selective degeneration of dopaminergic neurons in the SN, thus mimicking the selective cell loss observed in PD-affected brains at the early stages of the disease [39–43]. Moreover, the absence of neuronal loss at the VTA, despite hα-syn overexpression in this region, confirms the selective vulnerability of nigral neurons, as previously reported [44, 67, 68]. The molecular mechanisms underlying this selective vulnerability of the SN neurons are not fully elucidated, but converging lines of evidence suggest for a possible implication of the dopamine-induced oxidative stress, the large neuronal arborization of the dopaminergic neurons and the excessive mitochondrial activity in these neuronal populations, which could be exacerbated by α-syn overexpression [69–72]. It is important to note that in PD-diseased brains, neuronal degeneration is not restricted to the SN, and it progresses with time and affects other cerebral regions at later stages [73]. Therefore, we speculate that neuronal degeneration may also progress in our model and affect other brain regions at later time points post-AAV injection.

### Neuronal degeneration in the substantia nigra is associated with the accumulation of pathological α-syn and the triggering of a neuroinflammatory response

Converging lines of evidence reported that neuronal loss in PD brains is commonly associated with the accumulation of pathological α-syn [3, 13]. Using pS129 immunohistochemistry, the most common approach to detect pathological α-syn in post-mortem tissue, we did not observe any signal in different brain regions, suggesting that pathological α-syn is absent or that the aggregates are not yet mature enough to stain for pS129, as this marker is associated with the late events of the aggregation process [51, 74]. Nevertheless, the use of specific antibodies raised against fibrillar and oligomeric forms of α-syn detected the accumulation of pathological α-syn in the midbrain of hα-syn overexpressing mice. Moreover, the use of RT-QuIC, an ultrasensitive approach to detect pathological α-syn by amplifying oligomeric α-syn seeds [75, 76], alongside with a filter retardation assay, showed a high seeding capacity of samples extracted from the SN of hα-syn overexpressing mice. Altogether, these observations demonstrate that the large-scale overexpression of hα-syn induced a discrete accumulation of pathological α-syn in the midbrain.

It is noteworthy that in our model, the overexpression of hα-syn was associated with an increase in reactive microglia and astrocytes, indicating an enhanced neuroinflammatory response, which is known to play a crucial role in the development of Parkinson’s disease (PD) [77–80]. Therefore, the presence of this reaction serves as an important criterion for a relevant PD model. Interestingly, reactive astrogliosis was detected throughout the brain, possibly due to widespread overexpression and stimulation by hα-syn or the direct accumulation of hα-syn in these cells [81]. However, microglial activation was primarily confined to the midbrain region and exhibited a correlation with neuronal loss. This finding aligns with clinical observations using positron emission tomography imaging, which have reported localized microgliosis in the basal ganglia and midbrain during the early stages of the disease [82, 83]. Moreover, although some studies have suggested that activated microglia is a response to neuronal loss in the midbrain [84], microgliosis observed in our model could be linked to α-syn overexpression and accumulation in the nigral neurons or directly in microglia [85, 86]. Of note, the fact that the neuroinflammation and the selective dopaminergic neurons were exclusively occurring in the midbrain confirmed a causative link between the two phenomena, as previously suggested [77, 87, 88]. Finally, to further confirm that our model faithfully recapitulates neuroinflammation observed in PD and other synucleinopathies, follow up studies should be conducted to assess the involvement of the peripheral immune system by the evaluation of infiltering T cells in different brain regions after widespread α-syn overexpression. Indeed, converging lines of evidence from post-mortem clinical observations [89–91] and α-syn-based animal models of PD [90, 92, 93] reported the presence of infiltrating peripheral adaptive immune cells, specifically CD3+, CD4 + and CD8 + T cells, in PD-diseased brains and in close proximity to microglia and astrocytes, suggesting their potential role in activating brain-resident immune cells and to promoting inflammation and neurodegeneration [90, 91].

## Conclusions

In summary, we have created a new rodent model of PD, after a unique and non-invasive systemic delivery of AAV particles overexpressing hα-syn. This model combines the advantages of the two commonly used approaches to generate hα-syn-based model of PD, α-syn Tg animal models and AAV-based overexpression models. This new model, mimicking the early stages of the PD, offers a unique opportunity to unveil the cellular and molecular events associated with PD pathogenesis and its progression and to develop new therapeutic strategies targeting α-syn for the treatment of PD and related disorders.

### Electronic supplementary material

Below is the link to the electronic supplementary material.

Supplementary Material 1

Supplementary Material 2

Supplementary Material 3

Supplementary Material 4

Supplementary Material 5

Supplementary Material 6

Supplementary Material 7

Supplementary Material 8

Supplementary Material 9

Supplementary Material 10

Supplementary Material 11

Supplementary Material 12

Supplementary Material 13

## Data Availability

The datasets used and/or analyzed during the current study are available from the corresponding author on reasonable request.

## List of abbreviations

α-syn: Alpha-synuclein
AAV: Adeno-associated virus
BBB: Blood-brain barrier
CNS: Central nervous system
CIP: Calf intestinal alkaline phosphatase
Ctx: Cortex
EPM: Elevated-plus maze
hα-syn: Human α-syn
Hip: Hippocampus
LBs: Lewy bodies
mα-syn: Mouse alpha-synuclein
mTurq: mTurquoise2
MWM: Morris water maze
Mono: Monomeric α-syn
PBS: Phosphate-buffered saline
PBS-T: PBS-Tween
PD: Parkinson?s disease
PFA: Paraformaldehyde
PFF: Pre-formed fibrils
pS129: Phosphorylated α-syn at the residue Ser129
RT-QuIC: Real-time quaking-induced conversion
SN: Substantia nigra
Str: Striatum
Tg: Transgenic
TH: Tyrosine hydroxylase
ThT: Thioflavin T
VTA: Ventral tegmental area
