# Supplementary figures and images for: Non-invasive systemic viral delivery of human alpha-synuclein mimics selective and progressive neuropathology of Parkinson’s disease in rodent brains
Source: Mol Neurodegener. 2023 Nov 27;18:91. doi: 10.1186/s13024-023-00683-8 (PMC10683293; doi:10.1186/s13024-023-00683-8)

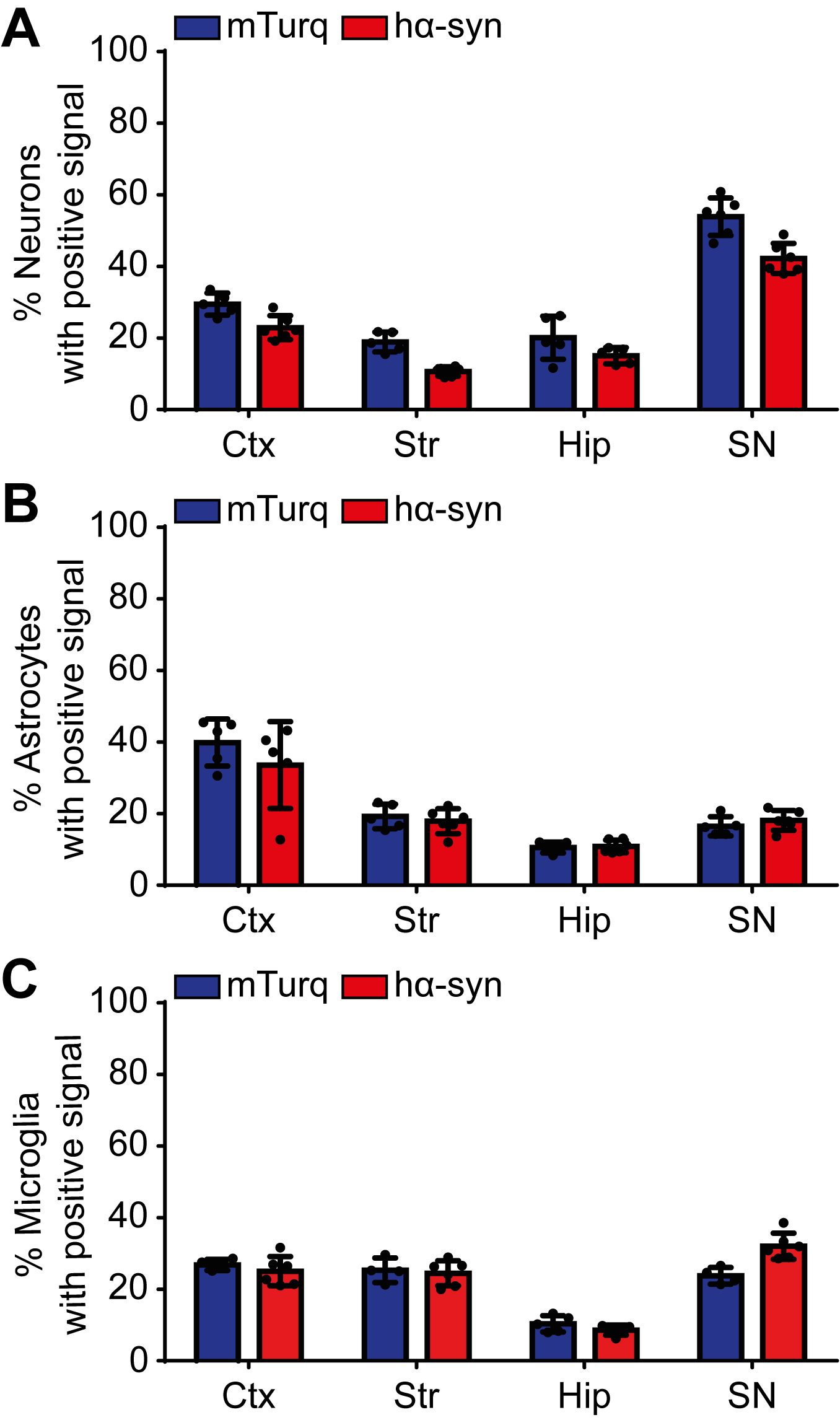

Supplement: Supplementary file 2 — Supplementary Material 2 [file 13024_2023_683_MOESM2_ESM.tif]

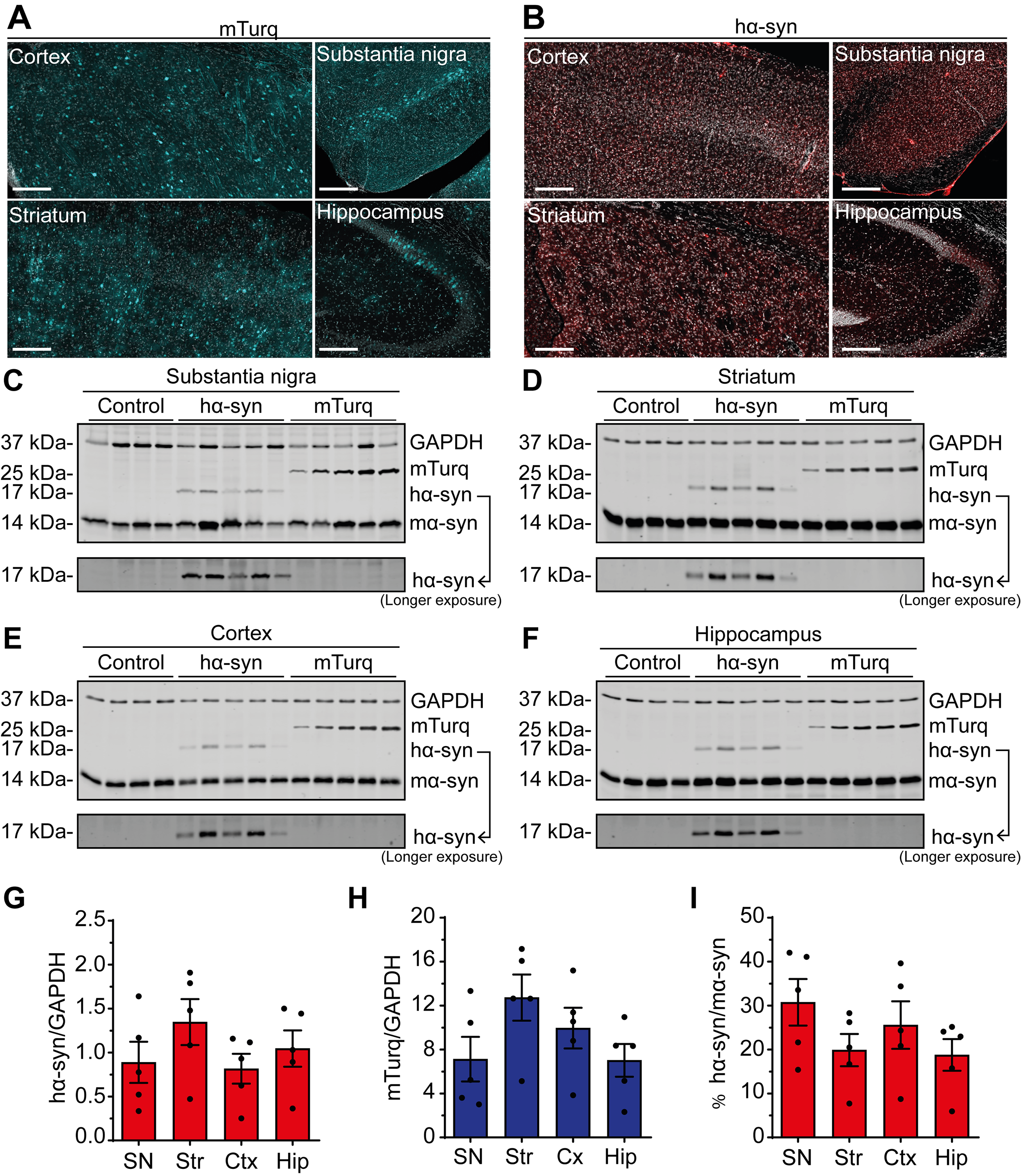

Supplement: Supplementary file 3 — Supplementary Material 3 [file 13024_2023_683_MOESM3_ESM.tif]

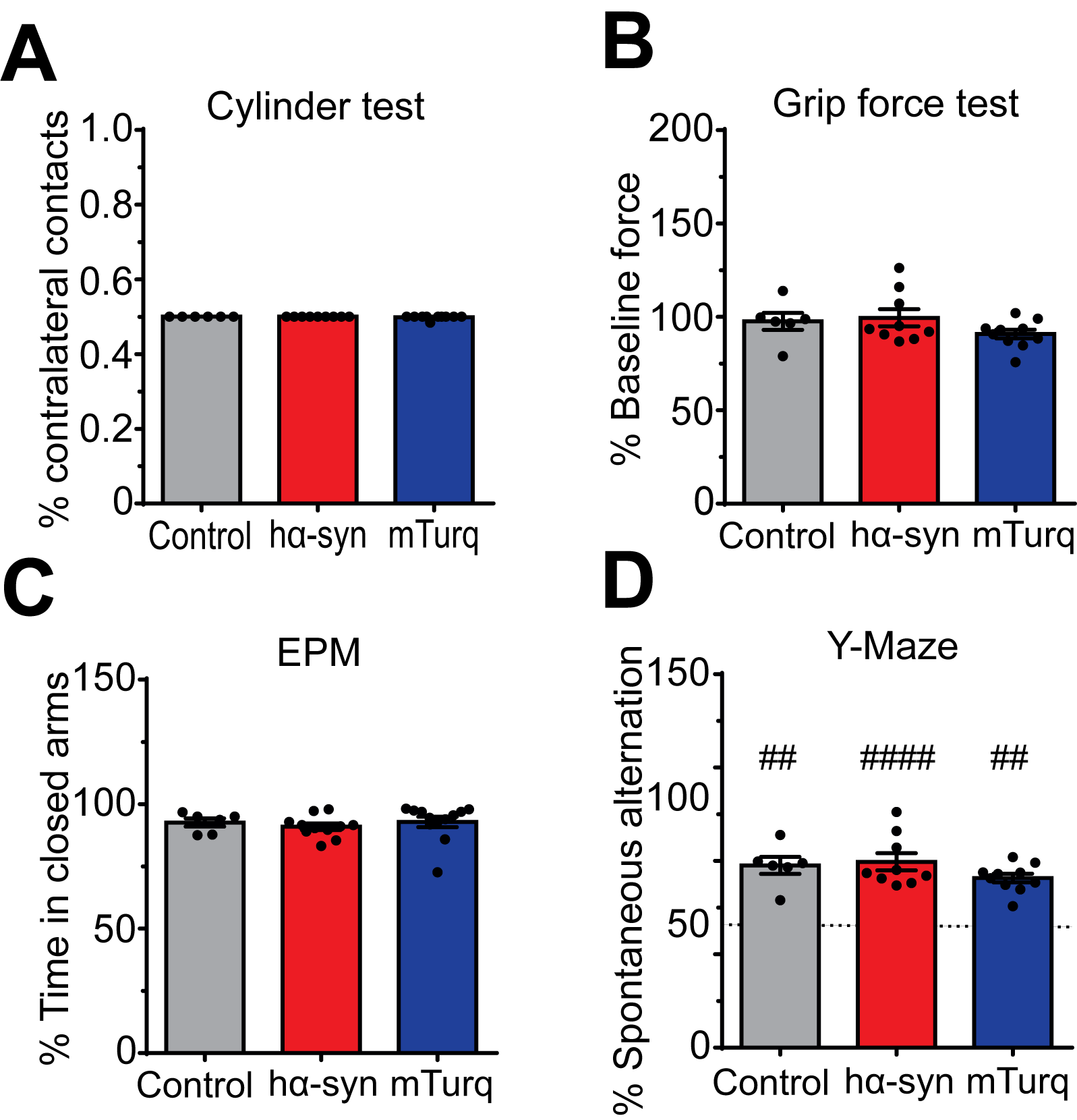

Supplement: Supplementary file 4 — Supplementary Material 4 [file 13024_2023_683_MOESM4_ESM.tif]

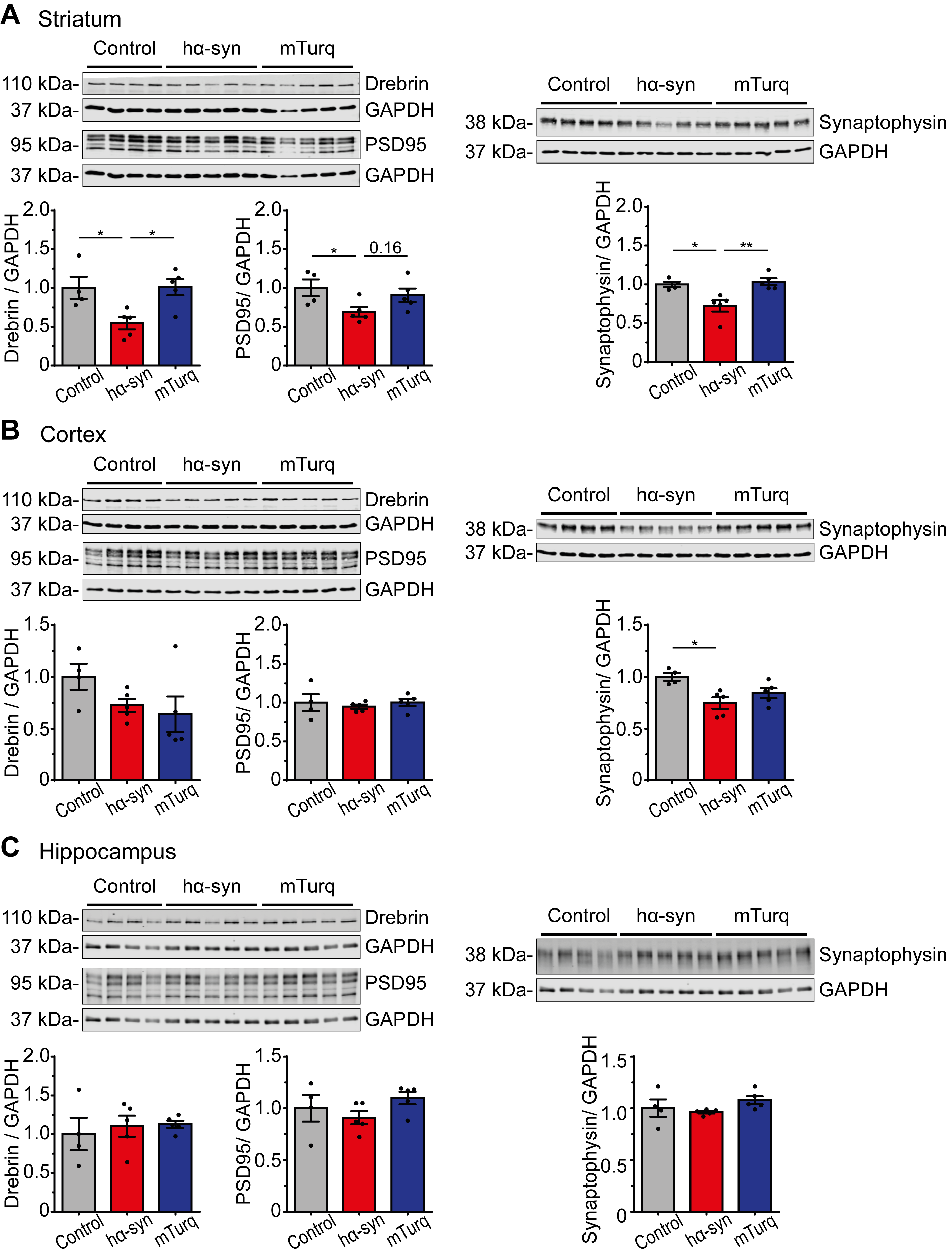

Supplement: Supplementary file 5 — Supplementary Material 5 [file 13024_2023_683_MOESM5_ESM.tif]

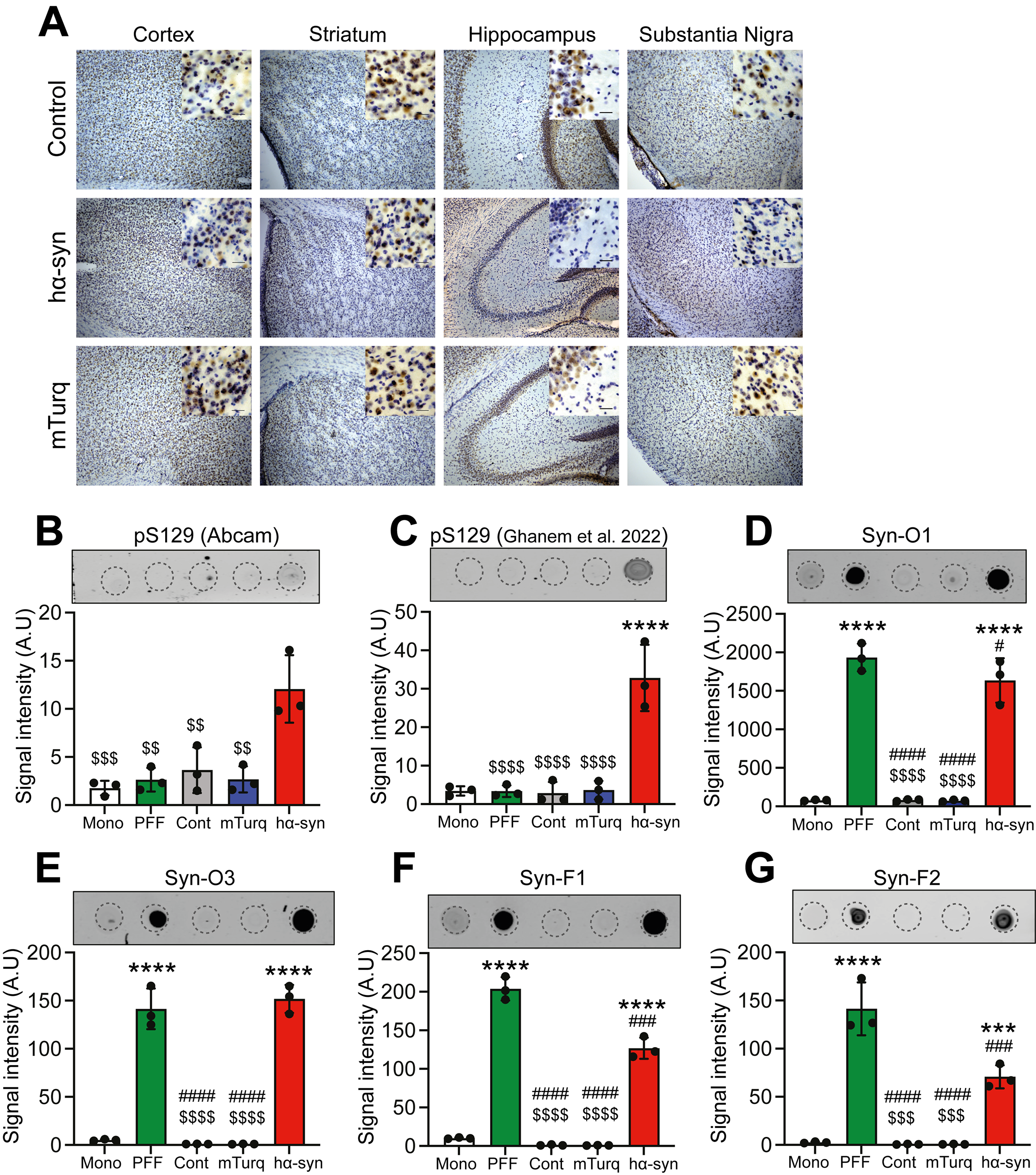

Supplement: Supplementary file 6 — Supplementary Material 6 [file 13024_2023_683_MOESM6_ESM.tif]

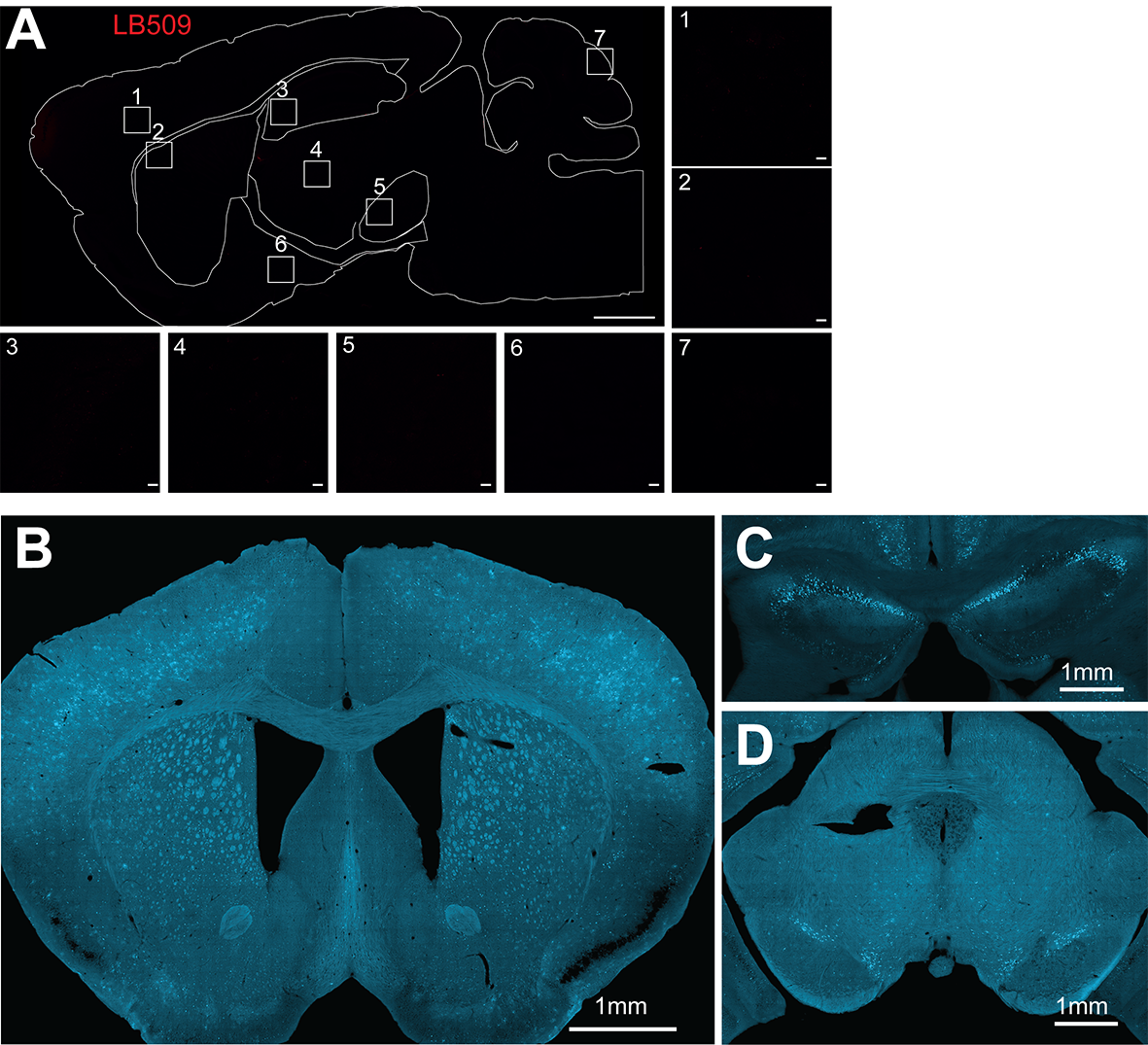

Supplement: Supplementary file 7 — Supplementary Material 7 [file 13024_2023_683_MOESM7_ESM.tif]

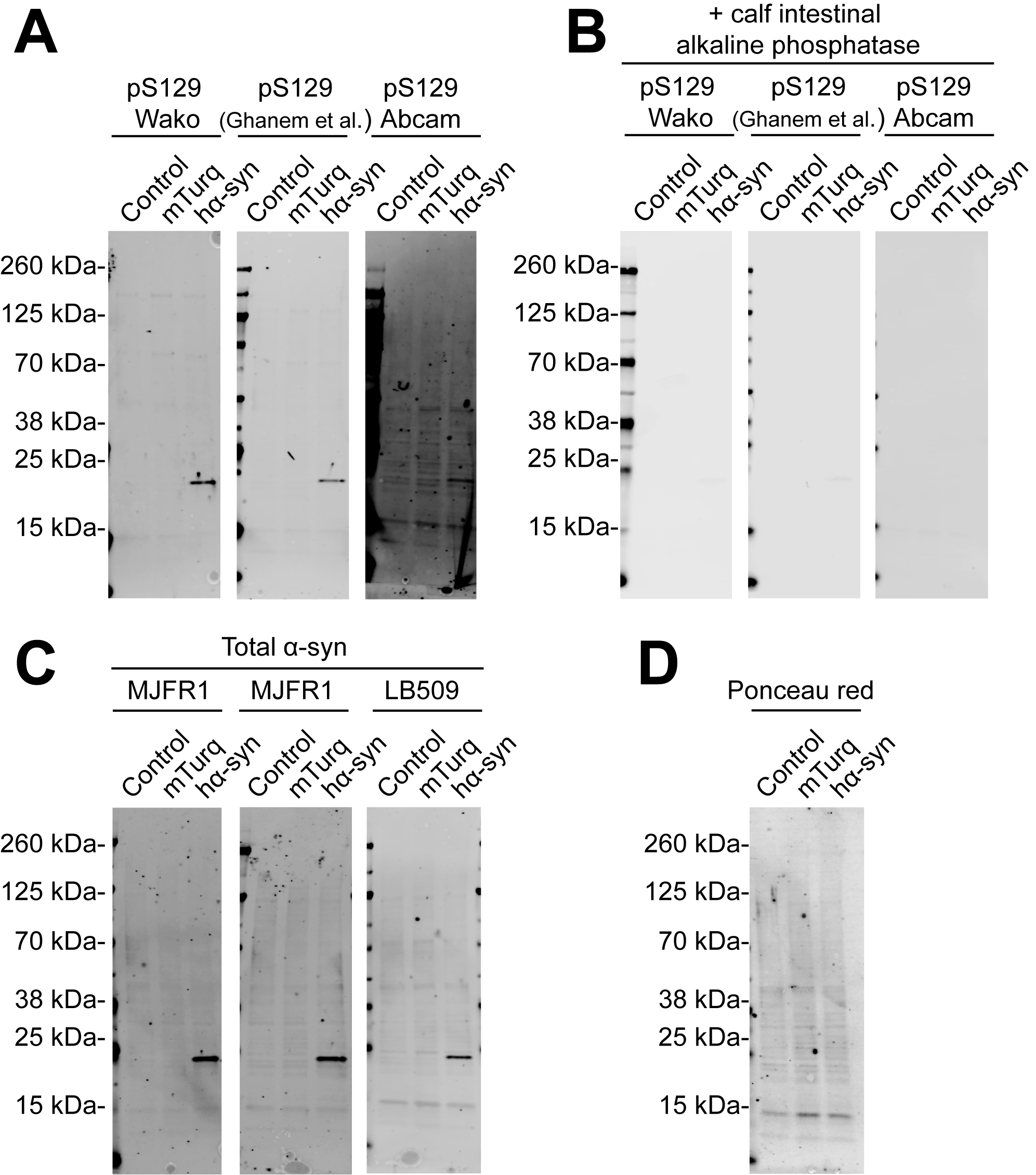

Supplement: Supplementary file 9 — Supplementary Material 9 [file 13024_2023_683_MOESM9_ESM.tif]
