# Supplementary material for: Non-invasive systemic viral delivery of human alpha-synuclein mimics selective and progressive neuropathology of Parkinson’s disease in rodent brains
Source: Mol Neurodegener. 2023 Nov 27;18:91. doi: 10.1186/s13024-023-00683-8 (PMC10683293; doi:10.1186/s13024-023-00683-8)
Supplement: Supplementary file 8 — Supplementary Material 8 [file 13024_2023_683_MOESM8_ESM.docx]

**Table 1: List of the antibodies used in this study**

| **Antigen/species** | **Antibody name /catalog number** | **Epitope** | **Concentration**  **immunoblotting** | **Concentration**  **IHC or IF** | **Source** |
| --- | --- | --- | --- | --- | --- |
| Primary antibodies | | | | | |
| α-syn/ mouse | Syn1 / 610787 | 15–123 | 1:1000 | 1:1000 | BD Laboratory  (Billerica, MA, USA) |
| Human α-syn/ mouse | LB509 / 180215 | 115-122 |  | 1:500 | ThermoFisher  Scientific (Waltham,  MA, USA) |
| α-syn/ rabbit | Syn FL-140 / sc-10717 | 65–91 | 1:1000 |  | Santa Cruz Biotech  (Dallas, TX, USA) |
| α-syn/ rabbit | Anti-Alpha-synuclein antibody [MJFR1]/ (ab138501) | 118-123 | 1:1000 |  | Abcam (Cambridge,  UK) |
| pS129 α-Syn/ mouse | WAKO / pSyn #64 | Phospho-Ser129 | 1:1000 | 1:2000 | WAKO (Richmond,  VA, USA) |
| pS129 α-Syn/rabbit | Anti-Alpha-synuclein (phospho S129) antibody [EP1536Y]/ Ab51253 | Phospho-Ser129 | 1:1000 |  | Abcam (Cambridge,  UK) |
| pS129 α-Syn/mouse | pS129 (Ghanem *et al*., 2022) | Phospho-Ser129 | 100 ng/ml |  | Ghanem *et al*., 2022 |
| Beta-actin/ mouse | β-actin clone BA3R / G043 | Beta-actin N-terminal peptide-KLH conjugates. | 1:10000 |  | Abm (Vancouver, BC, Canada) |
| Drebrin/mouse | Drebrin clone MX823/612,128 | C-terminal 632–649  coupled to KLH | 1:1000 |  | Progen (Heidelberg, Germany) |
| GAPDH/ mouse | GAPDH loading control/G041 | – | 1:2500 |  | Abm (Vancouver, BC, Canada) |
| GFP/ rabbit | GFP/A-6455 | Full length protein | 1:5000 |  | ThermoFisher  Scientific (Waltham,  MA, USA) |
| Non-phosphorylated α-syn/ mouse | 4B1 | 125-133 | 100 ng/ml |  | Ghanem *et al*., 2022 |
| Full-length α-syn aggregates /mouse | Syn-O2 |  | 100 ng/ml |  | Ghanem *et al*., 2022 |
| Full-length α-syn aggregates /mouse | Syn-O1 |  | 100 ng/ml |  | Ghanem *et al*., 2022 |
| Full-length α-syn aggregates /mouse | Syn-O3 |  | 100 ng/ml |  | Ghanem *et al*., 2022 |
| Full-length α-syn aggregates /mouse | Syn-F1 |  | 100 ng/ml |  | Ghanem *et al*., 2022 |
| Full-length α-syn aggregates /mouse | Syn-F2 |  | 100 ng/ml |  | Ghanem *et al*., 2022 |
| GFAP/ rabbit | anti-Glial Fibrillary Acidic Protein / Z0334 | Full length protein |  | 1:800 | Dako (Santa Clara, CA, USA) |
| GFAP/ mouse | Anti-Glial Fibrillary Acidic Protein  clone GA5/ MAB360 | Full length protein |  | 1:800 | Millipore (Temecula,  CA, USA) |
| Iba1/ rabbit | Anti-Ionized calcium binding adaptor molecule 1/ 019-19741 | Synthetic peptide (Iba1 C-terminal sequence) |  | 1:750 | WAKO (Richmond,  VA, USA) |
| mCherry/ rabbit | Anti-mCherry antibody/ AB167453 | mCherry | 1:1000 | 1:1000 | Abcam (Cambridge,  UK) |
| NeuN/ mouse | Anti-NeuN Antibody, clone A60 / MAB377 |  |  | 1:1000 | Millipore (Temecula,  CA, USA) |
| PSD95/mouse | PSD95 clone K28/43/75–028 | 77–299 (PDZ  domains 1 and 2) | 1:1000 UC |  | Davis/NIH NeuroMab Facility (Davis, CA, USA) |
| TH/ mouse | Anti-Tyrosine Hydroxylase  clone LNC1 /MAB318 | Recognizes an epitope on the outside of the regulatory N-terminus |  | 1:1000 | Millipore (Temecula,  CA, USA) |
| Synpato-physin/  rabbit | Synaptophysin/PA1–1043 | 253–272 1:50000– | 1:25000 |  | Invitrogen (Waltham, MA, USA) |
| Secondary antibodies | | | | | |
| IRDye 680RD Goat anti-Rabbit IgG Secondary Antibody | 680RD-conjugated goat anti-rabbit/ 926–68071 |  | 1:20000 |  | LI-COR Biosciences  (Lincoln, NE, USA) |
| IRDye 800CW Goat  anti-Rabbit IgG  Secondary Antibody | 800CW-conjugated goat anti-rabbit/ 926–32211 |  | 1:20000 |  | LI-COR Biosciences  (Lincoln, NE, USA) |
| IRDye 680RD Goat anti-Mouse IgG Secondary Antibody | 680RD-conjugated goat anti-mouse/ 926–68070 |  | 1:20000 |  | LI-COR Biosciences  (Lincoln, NE, USA) |
| IRDye 800CW Goat  anti-Mouse IgG  Secondary Antibody | 800CW-conjugated goat anti-mouse/ 926–32210 |  | 1:20000 |  | LI-COR Biosciences  (Lincoln, NE, USA) |
| Goat Anti-Mouse IgG Antibody (H+L), Biotinylated | Biotinylated Goat Anti-mouse/ BA-9200 |  |  | 1:500 | Vector Laboratories  (Burlingame, CA, USA) |
| Alexa Fluor 488 goat anti-rabbit (H+L) | Alexa Fluor 488 goat anti-rabbit/ A-11008 |  |  | 1:1000 | Invitrogen (Waltham,  MA, USA) |
| Alexa Fluor 633 goat anti-rabbit (H+L) | Alexa Fluor 633 goat anti-rabbit/ A21071 |  |  | 1:1000 | Invitrogen (Waltham,  MA, USA) |
| Alexa Fluor 488 goat anti-mouse (H+L) | Alexa Fluor 488 goat anti-mouse/ A-11029 |  |  | 1:1000 | Invitrogen (Waltham,  MA, USA) |
| Alexa Fluor 633 goat anti-mouse (H+L) | Alexa Fluor 633 goat anti-mouse/  A-21052 |  |  | 1:1000 | Invitrogen (Waltham,  MA, USA) |
| Cy™3 AffiniPure Goat Anti-Rabbit IgG (H+L) | 111-165-003 |  |  | 1:1000 | Jackson ImmunoResearch Laboratories, Inc  872 West Baltimore Pike  West Grove, Pennsylvania 19390  USA |
