# Supplementary material for: Non-invasive systemic viral delivery of human alpha-synuclein mimics selective and progressive neuropathology of Parkinson’s disease in rodent brains
Source: Mol Neurodegener. 2023 Nov 27;18:91. doi: 10.1186/s13024-023-00683-8 (PMC10683293; doi:10.1186/s13024-023-00683-8)
Supplement: Supplementary file 10 — Supplementary Material 10 [file 13024_2023_683_MOESM10_ESM.pdf]

Figure 1C: Substantia nigra

GAPDH / mTurq / hα-syn & mα-syn

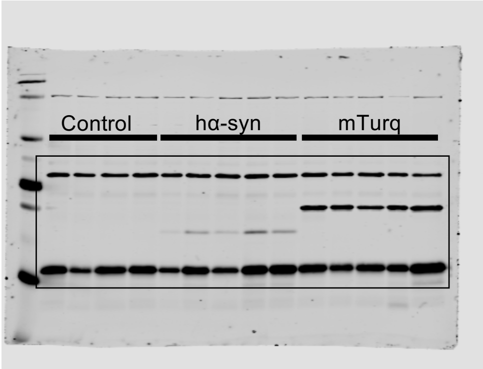

GAPDH
mTurq
hα-syn
mα-syn

hα-syn (longer exposure)

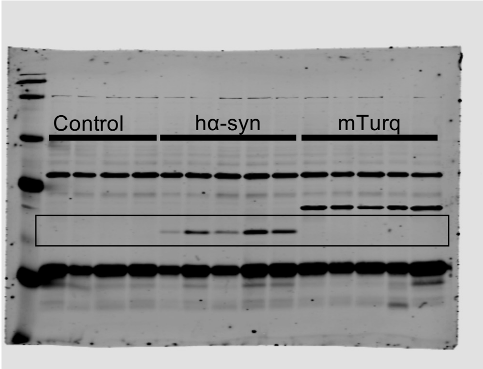

hα-syn

Figure 1D: Striatum

GAPDH / mTurq / hα-syn & mα-syn

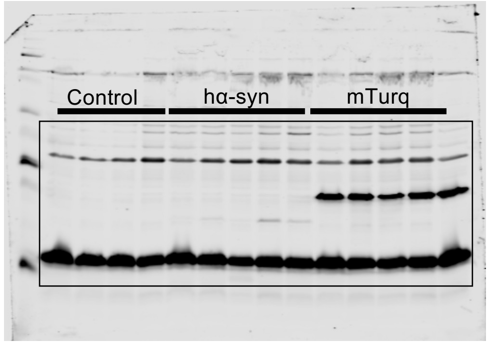

GAPDH
mTurq
hα-syn
mα-syn

hα-syn (longer exposure)

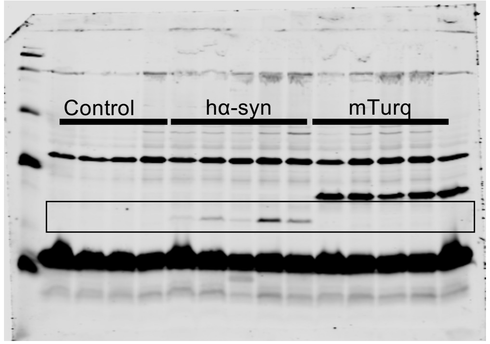

hα-syn

Boxed images were used in the main figure.

Visualization and quantification were carried out with the LI-COR Odyssey scanner and software (LI-COR Lincoln, NE, USA).

Figure 1E: Cortex

GAPDH / mTurq / hα-syn & mα-syn

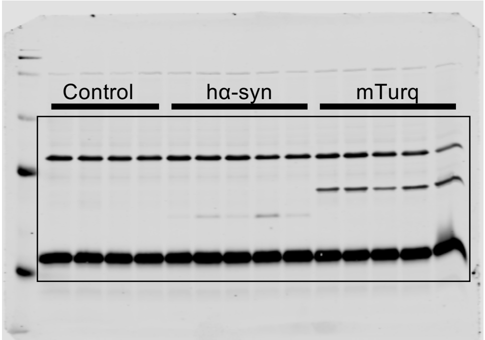

GAPDH
mTurq
hα-syn
mα-syn

hα-syn (longer exposure)

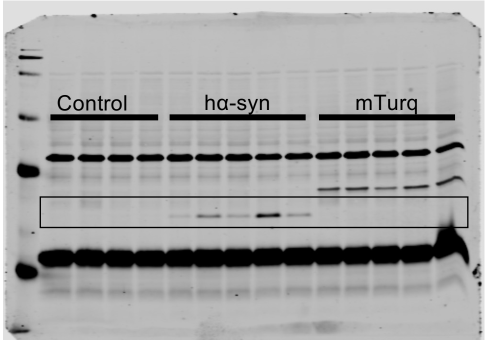

hα-syn

Figure 1F: Hippocampus

GAPDH / mTurq / hα-syn & mα-syn

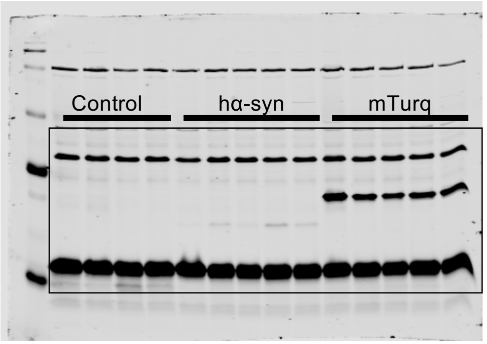

GAPDH
mTurq
hα-syn
mα-syn

hα-syn (longer exposure)

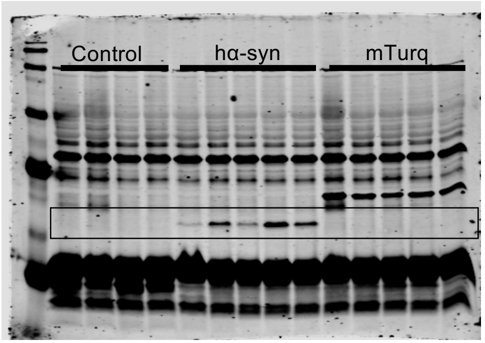

hα-syn

Boxed images were used in the main figure.

Visualization and quantification were carried out with the LI-COR Odyssey scanner and software (LI-COR Lincoln, NE, USA).

Figure 4

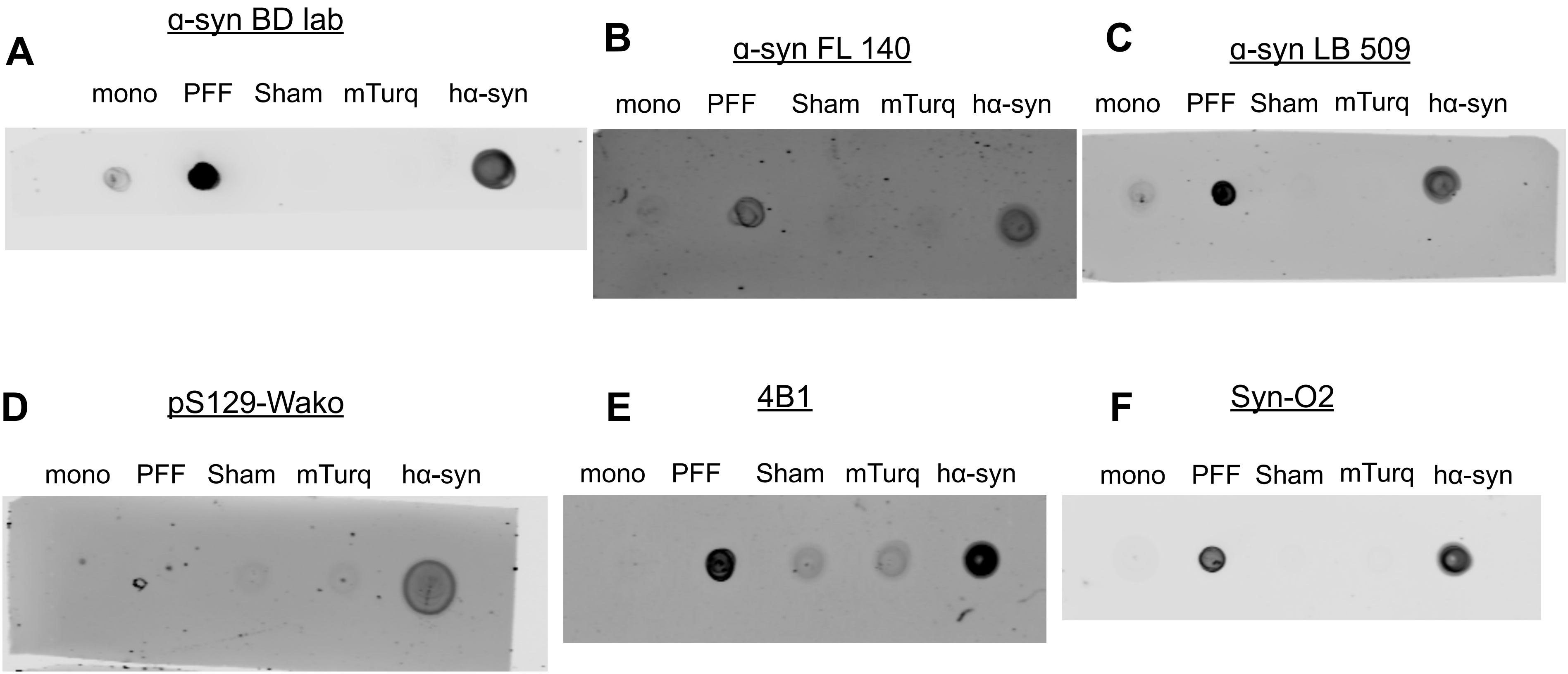

Abbreviations:
mono= α-syn monomers
PFF= Pre-formed Fibrils

Visualization and quantification were carried out with the LI-COR Odyssey scanner and software (LI-COR Lincoln, NE, USA).

Figure 4 H

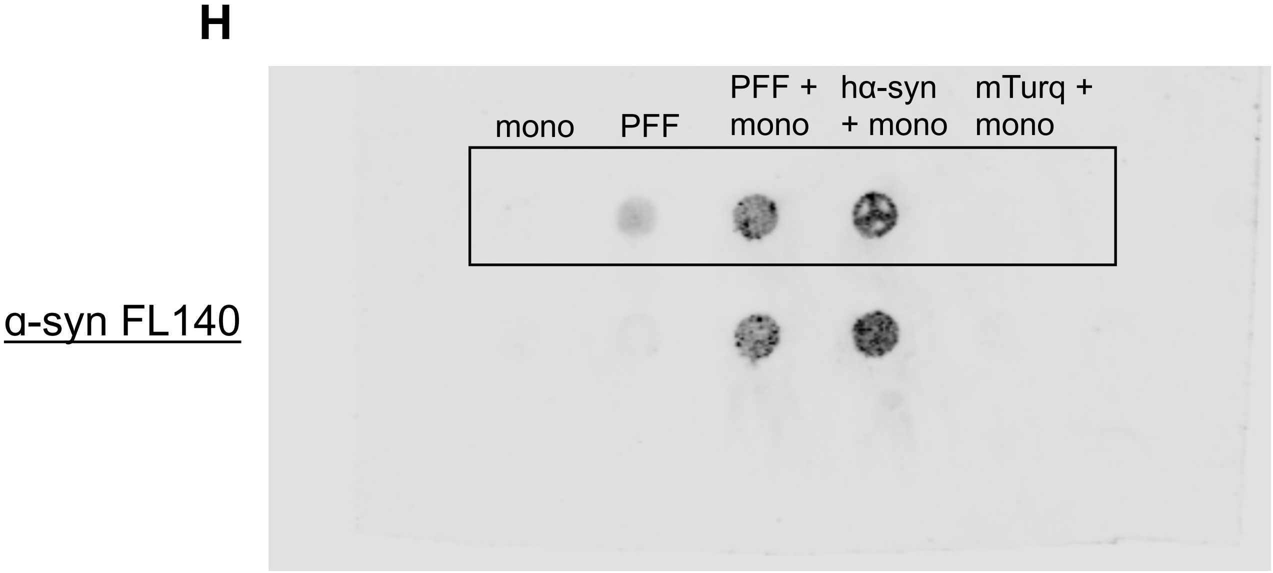

Suppl Figure 3C: Substantia nigra

GAPDH / mTurq / hα-syn & mα-syn

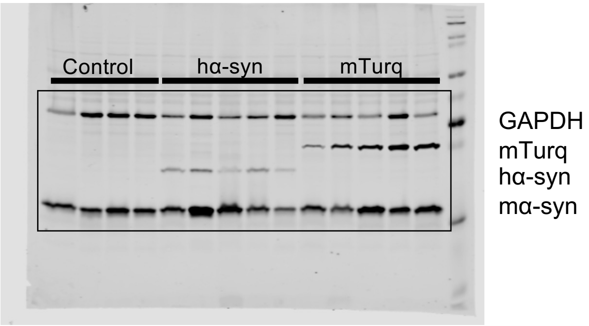

hα-syn (longer exposure)

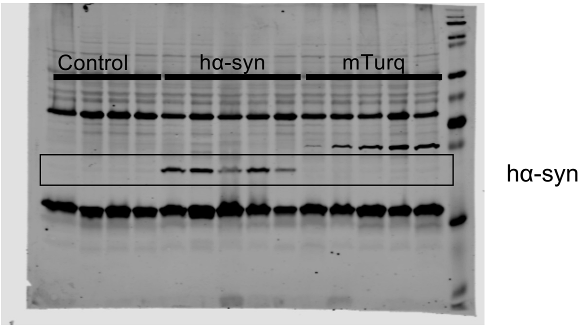

Suppl Figure 3D: Striatum

GAPDH / mTurq / hα-syn & mα-syn

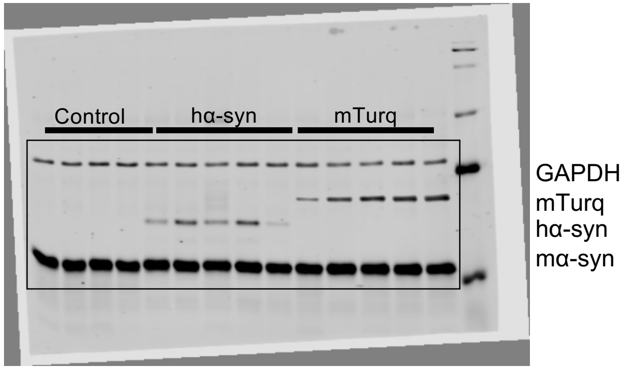

hα-syn (longer exposure)

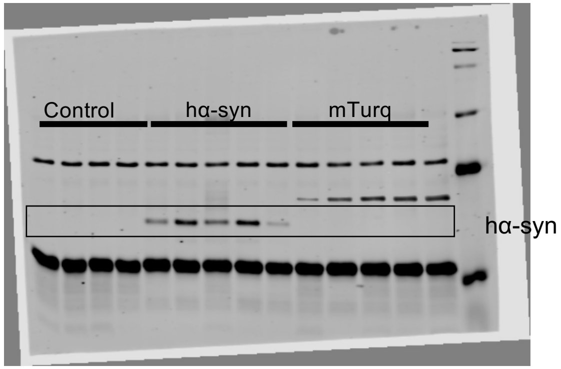

Boxed images were used in the main figure.

Visualization and quantification were carried out with the LI-COR Odyssey scanner and software (LI-COR Lincoln, NE, USA).

Suppl Figure 3E: Cortex

GAPDH / mTurq / hα-syn & mα-syn

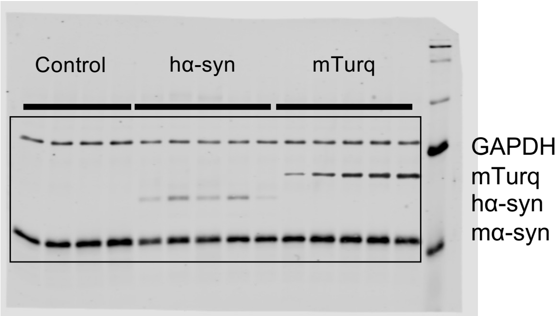

hα-syn (longer exposure)

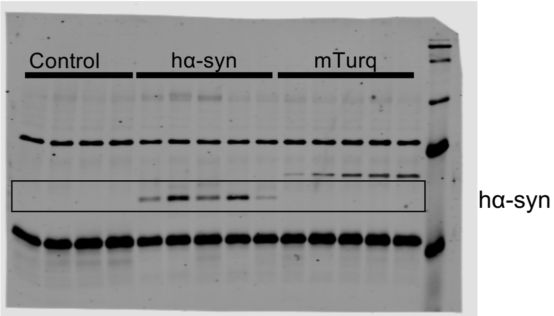

Suppl Figure 3F: Hippocampus

GAPDH / mTurq / hα-syn & mα-syn

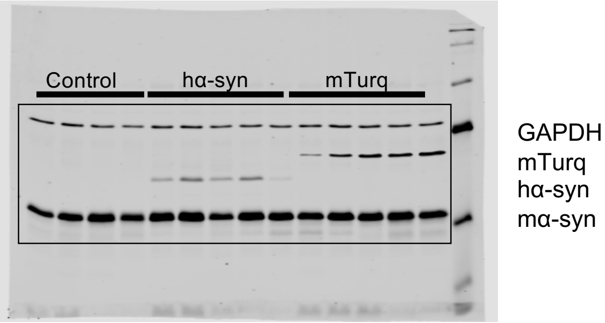

hα-syn (longer exposure)

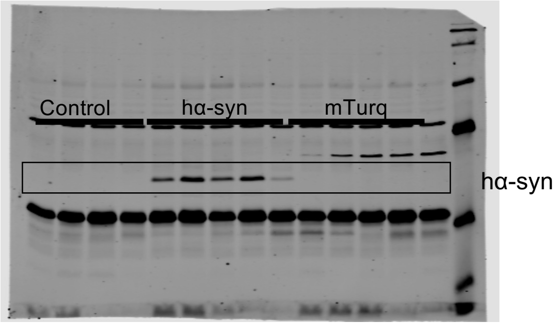

Boxed images were used in the main figure.

Visualization and quantification were carried out with the LI-COR Odyssey scanner and software (LI-COR Lincoln, NE, USA).

Suppl Figure 5A: Striatum

Drebrin / GAPDH

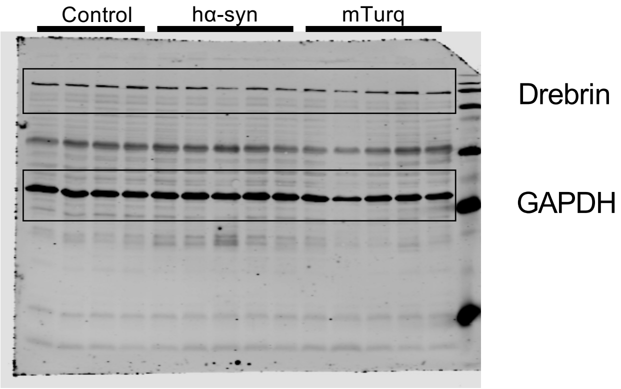

PSD95

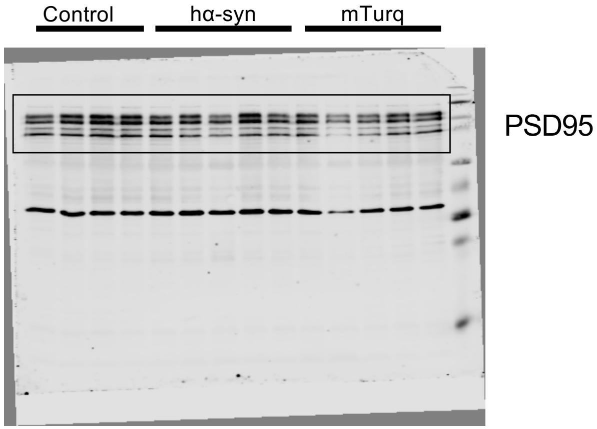

Synaptophysin

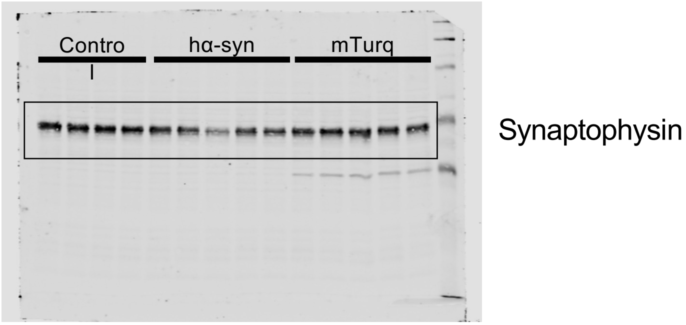

GAPDH

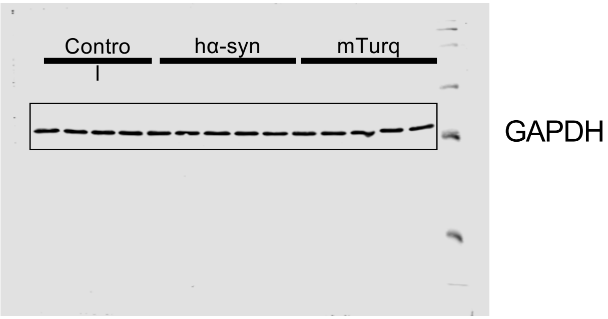

Boxed images were used in the main figure.

Visualization and quantification were carried out with the LI-COR Odyssey scanner and software (LI-COR Lincoln, NE, USA).

Suppl Figure 5B : Cortex

GAPDH

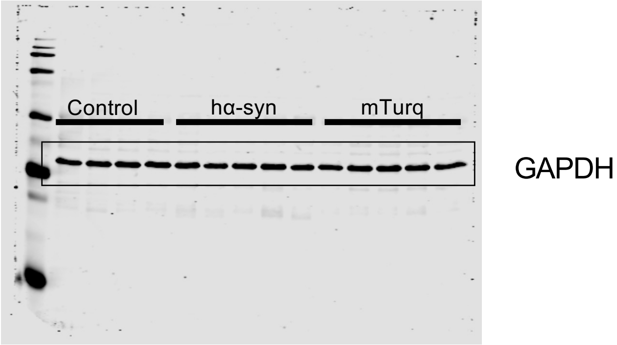

Drebrin

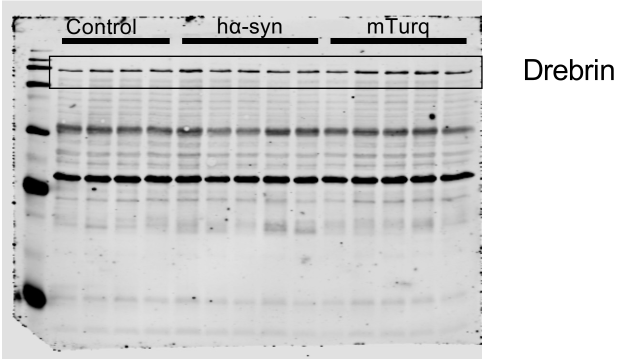

Synaptophysin

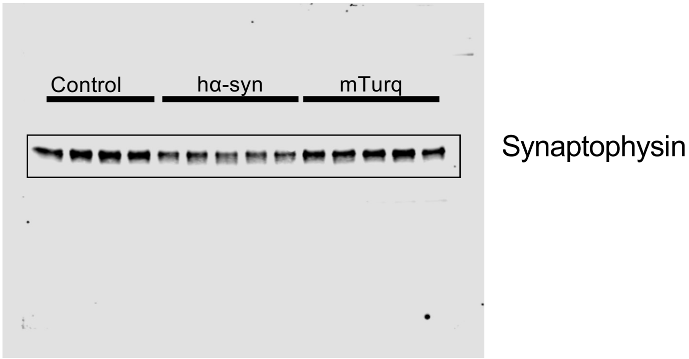

PSD95/GAPDH

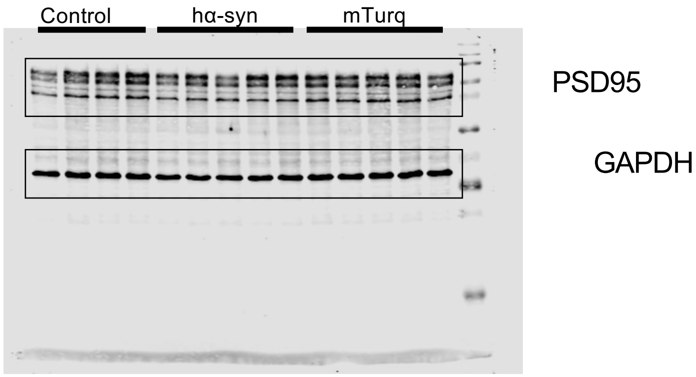

Boxed images were used in the main figure.

Visualization and quantification were carried out with the LI-COR Odyssey scanner and software (LI-COR Lincoln, NE, USA).

Suppl Figure 5C : Hippocampus

GAPDH

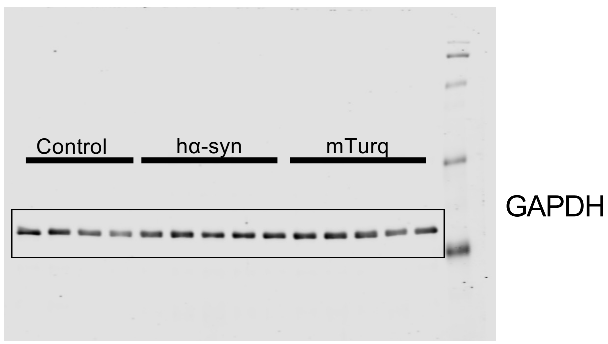

Drebrin

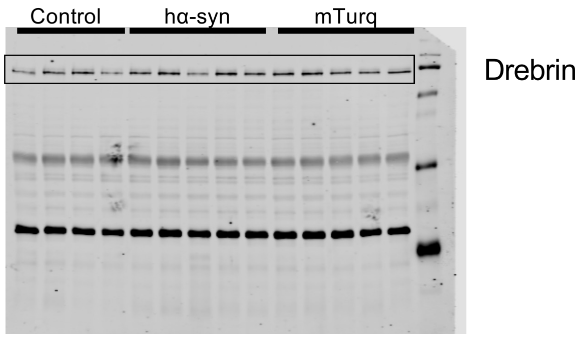

Synaptophysin

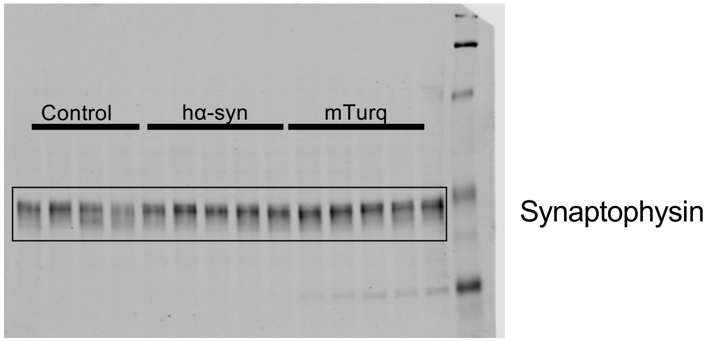

PSD95

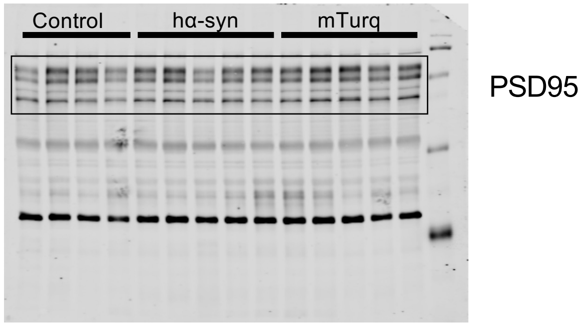

Boxed images were used in the main figure.

Visualization and quantification were carried out with the LI-COR Odyssey scanner and software (LI-COR Lincoln, NE, USA).

Suppl Figure 6

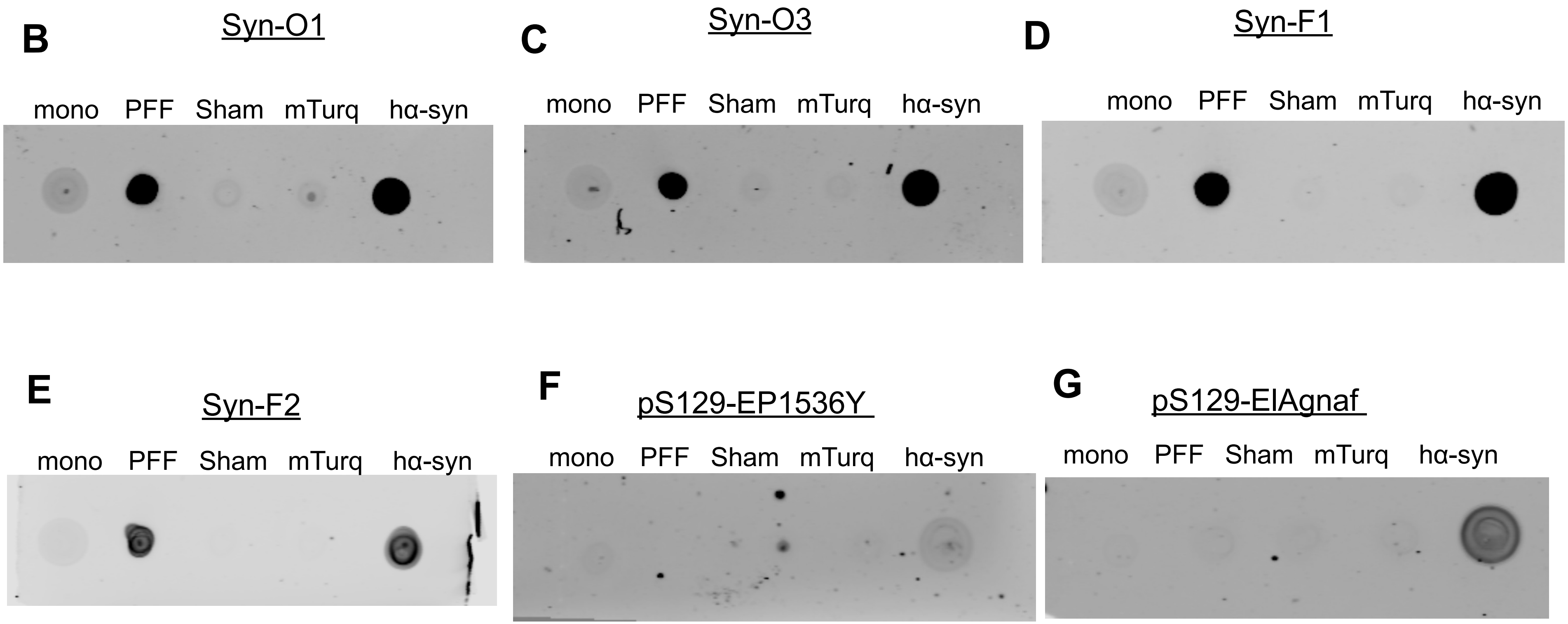

Abbreviations:
mono= α-syn monomers
PFF= Pre-formed Fibrils

Visualization and quantification were carried out with the LI-COR Odyssey scanner and software (LI-COR Lincoln, NE, USA).
