# Supplementary material for: Non-invasive systemic viral delivery of human alpha-synuclein mimics selective and progressive neuropathology of Parkinson’s disease in rodent brains
Source: Mol Neurodegener. 2023 Nov 27;18:91. doi: 10.1186/s13024-023-00683-8 (PMC10683293; doi:10.1186/s13024-023-00683-8)
Supplement: Supplementary file 11 — Supplementary Material 11 [file 13024_2023_683_MOESM11_ESM.docx]

**Suppl. Figure titles and legends**

**Suppl. Fig. 1:** **Unilateral injection of AAV-PHP.eB results in transgene expression in the two hemisphere 2-weeks post-AAV delivery**. (**A**) Representative confocal microscopy images of sagittal brain slices of control mice (scale bar=1 mm). Numbered boxes represent zoomed images of the different brain regions: (1) cortex, (2) striatum, (3) hippocampus, (4) thalamus, (5) midbrain, (6) hypothalamus, and (7) cerebellum (scale bar=10 µm). (**B-D**) Representative confocal images of coronal brain slices illustrating the bilateral pattern of mTurq expression in (**B**) the striatum and cortex, (**C**) the hippocampus, and (**D**) the midbrain. (Scale bar=1 mm).

**Suppl. Fig. 2: AAV-PHP.eB induces differential transgenes expression in different brain cell types 2-weeks post-AAV delivery.** (**A-C**) Quantification of the percentage of (**A**) neurons, (B) astrocytes, and (**C**) microglia expressing mTurq or hα-syn. The data are expressed as the means ± s.e.m. (n=6 mice per experimental condition).

**Suppl. Fig. 3: The systemic delivery of AAV-PHP.eB particles results in stable expression of the transgenes in the brain 3-months post-AAV injection.** (**A-B**) Representative confocal images of coronal brain slices of different brain regions (cortex, SN, striatum, and hippocampus) of mice overexpressing (**A**) mTurq or (**B**) hα-syn (scale bar=1 mm). (**C-D**) Western blot analysis of hα-syn, mα-syn and mTurq protein levels in the soluble protein fraction from the (**C**) substantia nigra, (**D**) striatum, (**E**) cortex, and (**F**) hippocampus. GAPDH was used as a loading control. (**G-H**) Quantification of the levels of (**G**) hα-syn and (**H**) mTurq in different brain regions normalized to GAPDH. (**I**) Ratio of hα-syn protein levels compared to endogenous mα-syn. The data are expressed as the means ± s.e.m. (n=4-5 mice per experimental condition). SN: substantia nigra, Str: striatum, Ctx: cortex, Hip: hippocampus. Full Western blots are shown in Suppl. Fig. 8.

**Suppl. Fig. 4: Overexpression of hα-syn does not affect the animals’ performances in certain motor and cognitive tests 3-months post-AAV delivery.** (**A**-**D**) Evaluation of animals’ performances using the (**A**) cylinder test, (**B**) grip force test, (**C**) elevated plus maze (EPM), and (**D**) Y-maze. The data are presented as the means ± s.e.m. (n=6-10 mice per experimental condition). One-sample t-test; (**D**) # p≤ 0.05, ## p ≤ 0.01 and #### p ≤ 0.0001 versus 50%.

**Suppl Fig. 5 Overexpression of hα-syn selective induces synaptic markers loss in the striatum 3-months post-AAV delivery.** (**A-C**) Western blot analysis of synaptic markers (drebrin, PSD95, and synaptophysin) in the soluble fraction from the (**A**) striatum, (**B**) cortex, and (**C**) hippocampus of mice. GAPDH was used as a loading control. The histograms present the protein levels of drebrin, PSD95 and synaptophysin expressed as synaptic marker/GAPDH normalized to the control group. The data are expressed as the means ± s.e.m. (n=4-5 mice per experimental condition). One-way ANOVA followed by Tukey’s multiple comparisons test; * p ≤ 0.05, ** p ≤ 0.01. Full Western blots are shown in Suppl. Fig. 8.

**Suppl. Fig. 6: Overexpression of hα-syn induces the formation of α-syn aggregates in the substantia nigra 3-months post-AAV delivery.** (**A**) Representative bright-field microscopy images of coronal brain slices illustrating pS129 (Wako) staining in different brain structures (cortex, striatum, hippocampus, and SN) (scale bar= 20 μm). (**B-G**) Dot blot analysis and quantification of 100 ng of protein prepared from the insoluble fraction of the SN of control, mTurq, and hα-syn mice (n=6 mice per experimental condition). Recombinant monomeric α-syn (Mono) and PFF were used as controls. Membranes were evaluated for protein expression against the following antibodies: (**B**) pS129 (Abcam), (**C**) pS129 (Ghanem *et al.* 2022), (**D**) Syn-O1, (**E**) Syn-O3, (**F**) Syn-F1, and (**G**) Syn-F1. The data are presented as the mean ± s.e.m. (n=3). One-way ANOVA followed by Tukey’s multiple comparisons test; *** p ≤ 0.001 **** p ≤ 0.0001 versus Mono; # p ≤ 0.05, ### p ≤ 0.001, #### p ≤ 0.0001 versus PFF; $$ p ≤ 0.01, $$$ p ≤ 0.001, $$$$ p ≤ 0.0001 versus hα-syn. Mono: α-syn monomer; PFF: pre-formed fibrils. Antibody detection: pS129: phosphorylated α-syn at S129; Syn-O1, Syn-O3, Syn-F1, and Syn-F2: α-syn aggregates. Full dot blots are shown in Suppl. Fig. 8.

**Suppl. Fig. 7: Validation of the specificity of pS129 antibodies. (A)** Western blot showing the detection of phosphorylated α-syn in the brain extract from mouse overexpressing h α-syn, but not in the control or the mTurq groups. (**B**) Western blot showing the loss of pS129 signal after membranes incubation with calf intestinal alkaline phosphatase. (**C**) Western blot showing the total hα-syn levels detected using MJFR1 and LB509 antibodies. (**D**) Ponceau red staining confirms the equal protein loading in the different experimental conditions.

**Suppl. Fig. 8: Full uncropped Western blots, dot blots, and filter retardation membranes for data shown in the manuscript.** Boxed images were used in the main figures. Visualization and quantification were carried out with the LI-COR Odyssey scanner and software (LI-COR Lincoln, NE, USA).
