# Supplementary material for: Non-invasive systemic viral delivery of human alpha-synuclein mimics selective and progressive neuropathology of Parkinson’s disease in rodent brains
Source: Mol Neurodegener. 2023 Nov 27;18:91. doi: 10.1186/s13024-023-00683-8 (PMC10683293; doi:10.1186/s13024-023-00683-8)
Supplement: Supplementary file 13 — Supplementary Material 13 [file 13024_2023_683_MOESM13_ESM.pdf]

UNIVERSITÉ LAVAL
Faculté de médicine
Département de médicine moléculaire

CHU de Québec
Centre de Recherche
Axe des Neurosciences

Abid Oueslati, Ph.D.
2705, boulevard Laurier. T-2-50
Québec (Québec) CANADA G1V 4G2
Phone: +1418-525-4444 Ext.49119
Mobile: +1418-264-5881

Quebec, November 8, 2023

Subject: manuscript# MOND-D-23-00057R1

Dear Dr. Mark Cookson

Please find enclosed a revised version of our manuscript entitled "Non-invasive systemic viral delivery of human alpha-synuclein mimics selective and progressive neuropathology of Parkinson’s disease in rodent brains”.

We would like to thank the reviewers for their thoughtful and constructive comments. All their comments have been addressed in the document Response to Reviewers, as well as in the revised version of the manuscript.

We hope that the editorial team will now find this much-improved version suitable for publication. Thank you for your time and kind consideration.

Sincerely,
Abid Oueslati
